# Amino alkynylisoquinoline and alkynylnaphthyridine compounds potently inhibit acute myeloid leukemia proliferation in mice

**DOI:** 10.1016/j.ebiom.2019.01.012

**Published:** 2019-01-25

**Authors:** N. Naganna, Clement Opoku-Temeng, Eun Yong Choi, Elizabeth Larocque, Elizabeth T. Chang, Brandon A. Carter-Cooper, Modi Wang, Sandra E. Torregrosa-Allen, Bennett D. Elzey, Rena G. Lapidus, Herman O. Sintim

**Affiliations:** a Purdue Institute for Drug Discovery, West Lafayette, IN 47907, USA; b Department of Chemistry, Purdue University, West Lafayette, IN 47907, USA; c Purdue University Center for Cancer Research, Purdue University, West Lafayette, IN 47907, USA; d Graduate Program in Biochemistry, University of Maryland, College Park, MD 20742, USA; e University of Maryland School of Medicine, Baltimore, MD, USA; f Department of Comparative Pathobiology, Purdue University, West Lafayette, IN 47907, USA

**Keywords:** Acute myeloid leukemia, FLT3-ITD (D835Y/F691L) inhibition, Src kinase inhibitors, FLT3 kinase inhibitors, Anti-leukemic effect

## Abstract

**Background:**

Acute myeloid leukemia (AML) remains one of the most lethal, rarely cured cancers, despite decades of active development of AML therapeutics. Currently, the 5-year survival of AML patients is about 30% and for elderly patients, the rate drops to <10%. About 30% of AML patients harbor an activating mutation in the tyrosine kinase domain (TKD) of Fms-Like Tyrosine kinase 3 (FLT3) or a FLT3 internal tandem duplication (FLT3-ITD). Inhibitors of FLT3, such as Rydapt that was recently approved by the FDA, have shown good initial response but patients often relapse due to secondary mutations in the FLT3 TKD, like D835Y and F691 L mutations.

**Methods:**

Alkynyl aminoisoquinoline and naphthyridine compounds were synthesized *via* Sonogashira coupling. The compounds were evaluated for their *in vitro* and *in vivo* effects on leukemia growth.

**Findings:**

The compounds inhibited FLT3 kinase activity at low nanomolar concentrations. The lead compound, HSN431, also inhibited Src kinase activity. The compounds potently inhibited the viability of MV4–11 and MOLM-14 AML cells with IC50 values <1 nM. Furthermore, the viability of drug-resistant AML cells harboring the D835Y and F691 L mutations were potently inhibited. *In vivo* efficacy studies in mice demonstrated that the compounds could drastically reduce AML proliferation in mice.

**Interpretation:**

Compounds that inhibit FLT3 and downstream targets like Src (for example HSN431) are good leads for development as anti-AML agents.

**Fund:**

Purdue University, Purdue Institute for Drug Discovery (PIDD), Purdue University Center for Cancer Research, Elks Foundation and NIH P30 CA023168.

## Graphical abstract

**Figure:**
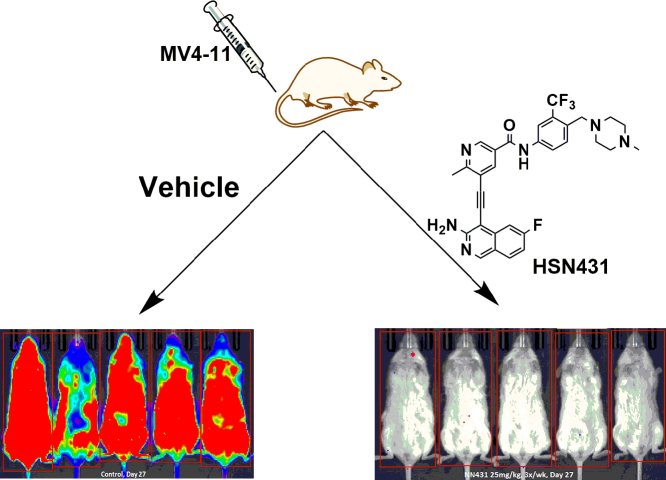

**Research in context**

**Evidence before this study**

FLT3 is a druggable target for about 30% of AML patients who harbor mutated FLT3 (ITD or TKD). A handful of FLT3 inhibitors evaluated in the clinic show good initial treatment response but patients ultimately relapse due to secondary mutations such as D835Y and F691 L, and other oncogenic activations. Hence therapeutic agents capable of inhibiting drug-resistantAML are desirable.

**Added value of this study**

In this study, we presented alkynyl aminoisoquinoline and naphthyridine compounds as novel inhibitors of FLT3 kinase activity as well as downstream kinases such as Src/Raf. Importantly, the compounds potently inhibited the growth of drug-resistantAML harboring the commonly observed D835Y and F691 L secondary mutations. The reported compounds are also efficacious *in vivo* (human AML xenograft models).

**Implications of all the available evidence**

This study provides new chemical entities that could be translated into therapeutics for relapsed AML patients who fail first/s generation FLT3 inhibitors, which are not very active against AML cells harboring D835 or F691 mutations.

Alt-text: Unlabelled Box

## 1 Introduction

Acute myeloid leukemia (AML) is a devastating disease, which still remains difficult to treat despite massive efforts by drug companies and academia to find durable cures [1]. The five-year survival rate for AML hovers around 30% and for elderly patients over 65 years, the five-year survival rate is unfortunately low (<10%) [[2], [3], [4]]. It is hoped that the survival rate for AML will soon improve due to the introduction of newer and more potent FLT3 inhibitors and advances made in allogeneic bone marrow transplantation. AML is a heterogenous disorder with an array of mutations that contribute differentially to prognosis [5]. About 30% of AML patients harbor a mutation in the Fms-Like Tyrosine Kinase 3 (FLT3), which makes the leukemia more aggressive [6,7]. Internal tandem duplication (ITD) in the juxtamembrane domain as well as tyrosine kinase domain (TKD) mutations, like those at residues D835 and F691, constitute FLT3 activating mutations [[8], [9], [10]]. When these mutations are present, FLT3 signaling bypasses the requirement of the FLT3 ligand for activation and hence becomes constitutively activated.

Midostaurin (Rydapt), a FLT3 tyrosine kinase inhibitor (TKI), was approved in 2017 [11,12] and other FLT3 TKIs are being evaluated in clinical trials [13]. Midostaurin is not effective as a single agent and it is administered in combination with chemotherapy [11,12]. The majority of the FLT3 inhibitors, which have been or are being evaluated in clinical trials show initial response, but patients often relapse with various FLT3 mutations (including secondary FLT3 mutations) and complete remission of AML is challenging [8,9,[14], [15], [16], [17]]. FLT3 D835Y/V [18] and F691 L [17] are common mutations, which often emerge during treatment and are resistant to many FLT3 TKIs. New-generation FLT3 inhibitors that could be used as a mono therapy and/or exhibiting potencies against mutated FLT3 (such as D835Y/V or F691 L) could improve AML survival rates.

**Fig. 1:**
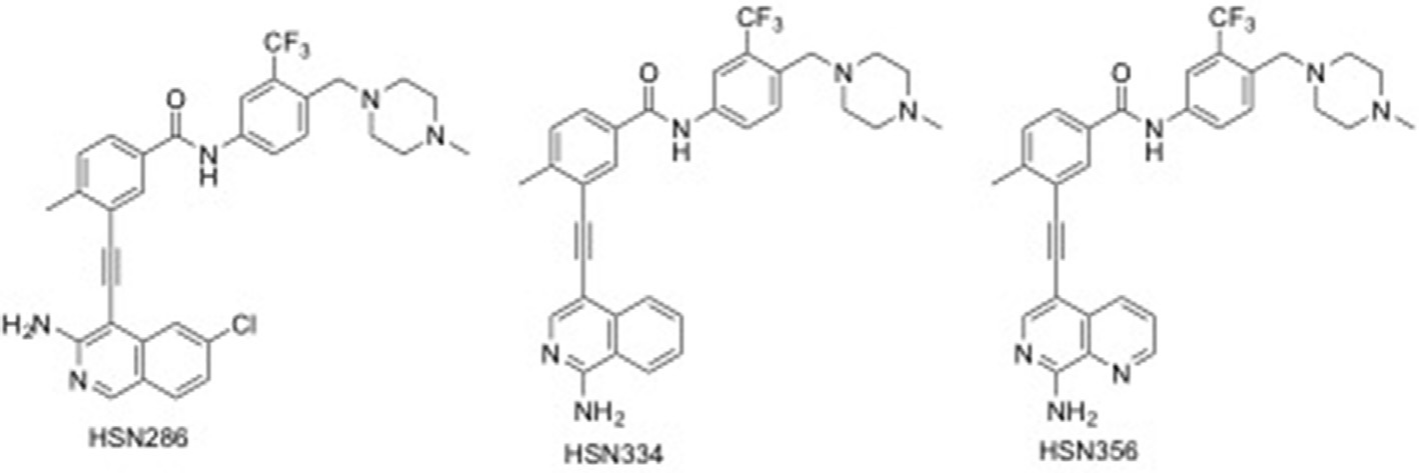
Dual FLT3/Src-kinase inhibitors reported by Larocque et al. [24].

Several kinases, which are downstream of FLT3, collaborate with constitutively active FLT3 (FLT3-ITD or FLT3 with mutation in the kinase domain, particularly the D835 or F691 mutations), to exacerbate AML [[19], [20], [21], [22]]. Src-family kinases play pivotal roles in microenvironment-induced resistance to FLT3 inhibition [23]. Therefore, dual inhibitors of FLT3-Src-family kinases could be effective in overcoming drug resistance. We recently reported that novel dual FLT3-Src-family kinase inhibitors (see Fig. 1) that contain alkynyl aminoisoquinoline moiety potently inhibited FLT3-ITD harboring AML cell lines, such as MV4–11 and MOLM-14, with single digit nanomolar or even sub-nanomolar half maximal inhibitory concentration (IC50) values *in vitro* [24]. The impressive *in vitro* efficacies of the alkynyl aminoisoquinoline and alkynyl aminonaphthyridine compounds against AML cell lines harboring FLT3-ITD prompted us to conduct an extensive structure-activity relationship (SAR) studies and to evaluate the efficacy of these compounds in mice. Here we present the SAR and corresponding efficacies of the second-generation alkynyl aminoisoquinoline and alkynyl aminonaphthyridine compounds.

## 2 Materials and methods

### 2.1 Chemicals, reagents and cell culture

Midostaurin was purchased from Sigma (St. Louis, MO, USA), crenolanib, quizartinib and ponatinib were purchased from Adooq Bioscience (Irvine, CA, USA). MV4–11 cell line was a kind gift from Dr. Mark Levis (Johns Hopkins University). MOLM-14 (FLT3-ITD, D835Y) and MOLM-14 (FLT3-ITD, F691 L) [25] were kind gifts from Dr. Neil Shah (UCSF). The cells were routinely maintained in Roswell Park Memorial Institute (RPMI) 1640 medium supplemented with 10% fetal bovine serum at 37 °C with 5% CO_2_.

### 2.2 Analysis of antiproliferative activities

Cells were seeded into 96-well tissue culture-treated plates at 2.0 × 10^4^ cells/mL for up to 24 h. DMSO stock solutions of compounds were serially diluted first in DMSO and subsequently into RPMI before being added to the cell cultures in triplicates, with 10 μM being the highest tested concentration. The plates were incubated for 72 h as above. The CellTitre-Blue assay (Promega, Madison WI) was then added to the cultures and incubated a further 4 h before the fluorescence measured following the manufacturers recommendations. The fluorescence data from compounds were normalized to that DMSO and the resulting data fitted to a non-linear regression equation to obtain IC50 using GraphPad Prism 5.0 Software (La Jolla, CA, USA).

To determine the effect of HSN431 on actual leukemia cell counts, MV4–11 cells were seeded into 96 well plates as above and treated with either DMSO vehicle, or HSN431 at the following concentrations: 0.1 nM, 0.2 nM, 0.5 nM, 1 nM, 2 nM, 5 nM, and 10 nM. Cells were incubated for 72 h, and then counted using trypan blue exclusion on the Countess automated cell counter (Life Technologies, Carlsbad, CA). Cell counts were performed in triplicates, and the averages, standard deviations and *t*-test statistical analyses were graphed using GraphPad Prism software (GraphPad, La Jolla, CA).

### 2.3 *In vitro* kinase assay

Cell-free kinase activity assays were performed at Reaction Biology Corporation. Briefly, kinases were incubated with substrates, 100 μM ATP and a 3-fold serial dilution of compounds starting at 1 μM. DMSO was used as control. For single-dose inhibition of FLT3-ITD and Src kinase activity, compounds were tested at 500 nM. Experiments were performed in duplicates. The concentrations of kinases and substrates used in the assays are indicated on Table S1. Kinase activity was quantified using the ADP-Glo™ Kinase Assay System (Promega, Madison, WI) following the manufacturer's recommendations.

### 2.4 *In vivo* efficacy studies

The *in vivo* efficacy of compounds was assessed in an orthotopic human AML model in NSG (NOD scid gamma, NOD-scid IL2Rg^null^) mice that were bred at University of Maryland Baltimore (mating pairs acquired from Jackson Laboratories; Maine). Female NSG or NRG (NOD-Rag1^null^ IL2rg^null^, NOD rag gammaR^null^, bred at Purdue University) mice (6–8 weeks old) were injected with 1 × 10^6^ MV4–11-luc cells, a gift from Dr. Sharyn Baker (Ohio State University) *via* intravenous injection into the lateral tail vein of restrained mice. Three days later, engraftment was assessed by imaging on a Xenogen IVIS-2 Imaging System (Alameda) after an intraperitoneal (IP) injection of 150 mg/kg luciferin. Mice were sorted into four or five groups (*n* = 5) so that mean intensity (*i.e.*, disease burden) was equal. Dosing started on day of sorting. Compounds were dissolved in 85% PBS (pH 7.4) buffer containing 10% DMSO and 5% Cremophore. The vehicle (buffer without compounds) was used as control. Mice were injected 3 to 5 days per week *via* IP injection. Mice were weighed on day of dosing and imaged once per week. The endpoint of each study was survival (mice were euthanized *via* AVMA-approved methods when they lost >20% of body weight or displayed hind limb paralysis). All mice were housed in a 12-h light/dark cycle with access to food and water *ad libitum*. Studies were performed with Institutional Animal Care and Use Committee (UMB and Purdue University) approval.

### 2.5 Western blot analysis

MV4–11 cultures were treated with 2 nM and 10 nM of compounds for 6 h and 48 h. Total protein extraction, gel separation, transfer and imaging of blots were performed as previously described [26]. The following primary antibodies from Cell Signaling Technologies (MA, USA) were used: Phospho-p70S6K (cat. no. 9205, RRID: AB_330944), p70S6K (cat. no. 9202, RRID: AB_331676), Phospho-p38 (cat. no. 9211, RRID: AB_331641), p38 (cat. no. 9212, RRID: AB_330713), c-Myc (cat. no. 9402, RRID: AB_2151827), Phospho-FLT3 (cat. no. 3461, RRID: AB_331060), FLT3 (cat. no. 3462, RRID: AB_10693420), Phospho-STAT5 (cat. no. 9359, RRID: AB_823649), STAT5 (cat. no. 9363, RRID: AB_2196923), Phospho-ERK (cat. no. 9101, RRID: AB_331646), ERK (cat. no. 4695, RRID: AB_390779), Phospho-AKT (cat. no. 9271), AKT (cat. no. 9272, RRID: AB_329825), Phospho-Src (cat. no. 2101, RRID: AB_331697), Src (cat. no. 2110, RRID: AB_2106058), Phospho-STAT3 (cat. no. 9145, RRID: AB_2491009), STAT3 (cat. no. 12640, RRID: AB_2629499). Mouse anti-β-Actin antibody was purchased from Sigma (MO, USA). A horseradish peroxidase-conjugated anti-rabbit or anti-mouse antibody (Cell Signaling Technologies) was used as secondary antibody. Scanned images were analyzed using image J software.

### 2.6 Cell cycle analysis and apoptosis

Cells seeded into T-25 flasks at a density of 8.0 × 10^4^ cells/mL and incubated overnight at 37 °C with 5% CO_2_. Each treatment was performed in triplicates, with three flasks per treatment each with enough volume for both assays at the applicable time points. Following the overnight incubation, cells were treated with either DMSO or different concentrations of HSN431 (as indicated) in triplicates, mixed well and incubated. At each time point (24 and 48 h), aliquots of 1.5 mL of cells were collected from each flask and placed into labeled FACS tubes (for Annexin V apoptosis) or labeled 15 cc conical tubes (for cell cycle) and centrifuged at 300 × *g* for 5 min. Cells were washed twice with 1× PBS.

For cell cycle, cell pellets were resuspended in 0.5 mL PBS in 15 cc conical tubes and fixed with 4.5 mL of 70% ethanol. Fixed cells were stored at −20 °C until analysis with the Propidium Iodide (PI) Flow Cytometry Kit (cat. no. ab139418, Abcam, Cambridge, MA, USA). Samples were analyzed in duplicates on a FACSCanto II flow cytometer (BD Bioscience, San Jose, CA, USA) and cell cycle distribution was analyzed using the Watson algorithm analysis in FlowJo software (Flowjo, LLC, Ashland, OR).

For Annexin V apoptosis, cell pellets were resuspended in 100 μL of 1× assay buffer and the binding of Annexin V and PI were measured by staining cells following the BD Pharmingen™ FITC Annexin V Apoptosis Detection Kit I (cat. no. 556547, BD Biosciences, San Jose, CA, USA) recommendations. Flow cytometry was performed on a a FACSCanto II flow cytometer (BD Bioscience, San Jose, CA, USA) and data analyzed using the FlowJo software (Flowjo, LLC, Ashland, OR).

### 2.7 Statistical analysis

Non-linear regression analysis of the cell-free *in vitro* kinase activity dose-response data were performed using GraphPad Prism 5.0 Software (GraphPad, La Jolla, CA, USA). Photon intensity on whole body and animal survival data were analyzed by students *t*-test (paired; two tailed) in GraphPad Prism 5.0 Software (CA, USA). Significant differences have been indicated as * (*P* ≤ .05), ** (*P* ≤ .01) or *** (*P* ≤ .001) according to the level of significance.

## 3 Results

### 3.1 Second generation compounds inhibit FLT3/Src kinase activity

**Fig. 2:**
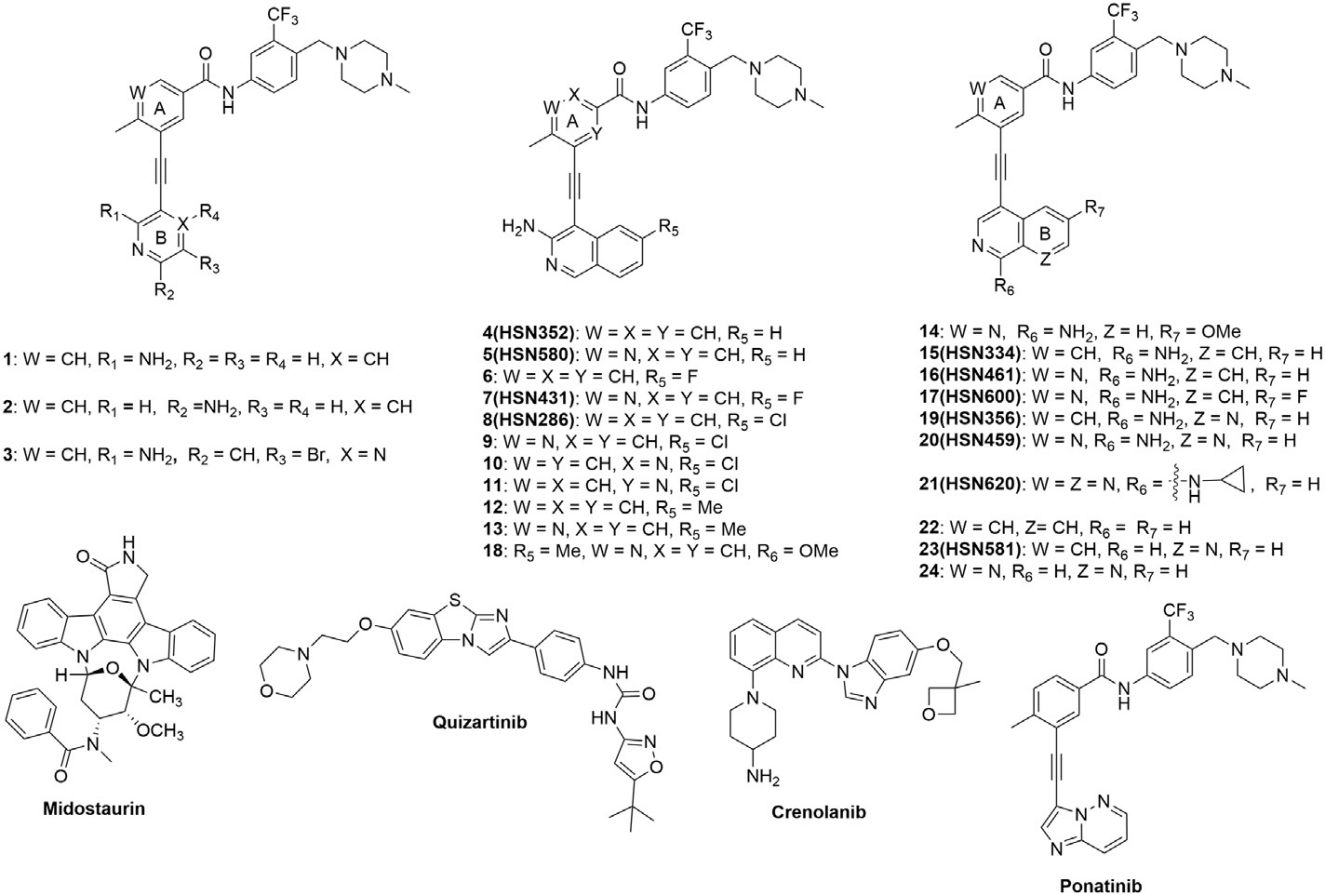
Alkynyl aminoisoquinoline and alkynyl naphthyridine compounds and other FLT3 inhibitors (midostaurin, quizartinib, crenolanib and ponatinib) evaluated in this study.

For the expanded SAR study, additional nitrogens were incorporated into rings A and B (see Fig. 2) of the first-generation compounds HSN286, HSN334 and HSN356 to improve aqueous solubility (lower LogP). In addition to the water-solubilizing effects of basic nitrogens, it has also been well documented that the introduction of the so-called “essential nitrogen” or nitrile moieties into drugs improves target engagement [27,28]. Fluorine has also been deemed a “magic” moiety that when incorporated into drugs could improve the potency as well as stability of the drugs [29]. Therefore, analogs which also contained fluorine were synthesized (compounds 6, 7 and 17; Fig. 2).

The synthesis of the second-generation FLT3-Src inhibitors (Fig. 2) were accomplished following a Sonogashira protocol previously reported by us, see SI [24]. With the compounds in hand, we evaluated their inhibitory activities against FLT3 and Src kinases at 500 nM concentrations using the Reaction Biology company kinase screening service (SI, Table S2). Compounds containing either monocyclic (aminopyridines) or bicyclic (amino isoquinoline or naphthyridine) moieties (in Fig. 2) potently inhibited (>90% inhibition of enzymatic activity at 500 nM) FLT3 and Src kinases. When the benzamide moiety in HSN286 (compound 8), HSN334 (compound 15) or HSN356 (Compound 19) was replaced with a nicotinamide to give compounds 9, 16 and 20, a significant increase (~1 log unit) in predicted aqueous solubility was achieved (compare cLogP of compound 8 (5.7) with compound 9 (4.4); compound 15 (4.9) with compound 16 (3.6) and compound 19 (4.0) with compound 20 (2.7)). The replacement of the benzamide moiety in HSN286 with a nicotinamide moiety (9) did not affect FLT3 or Src inhibition. Compound 9 inhibited the proliferation of FLT3-ITD-harboring MV4–11 cells with a similar potency to HSN286 (both compounds inhibited MV4–11 cells with IC50 value of ~0.5 nM). However, the replacement of the benzamide moiety in HSN286 with picolinamide moieties (HSN286 to 10 or 11), drastically affected the inhibition of FLT3 enzymatic activity (at 500 nM, HSN286 inhibited FLT3 enzymatic activity by 97% whereas the picolinamide analogs, compounds 10 and 11 inhibited FLT3 at 75% and 43% respectively, see Table S2). Therefore, we opted to make the nicotinamide derivatives HSN461 (compound 16), HSN459 (compound 20), compounds 5, 7, 13, 14, 18, 21 and 24.

**Fig. 3:**
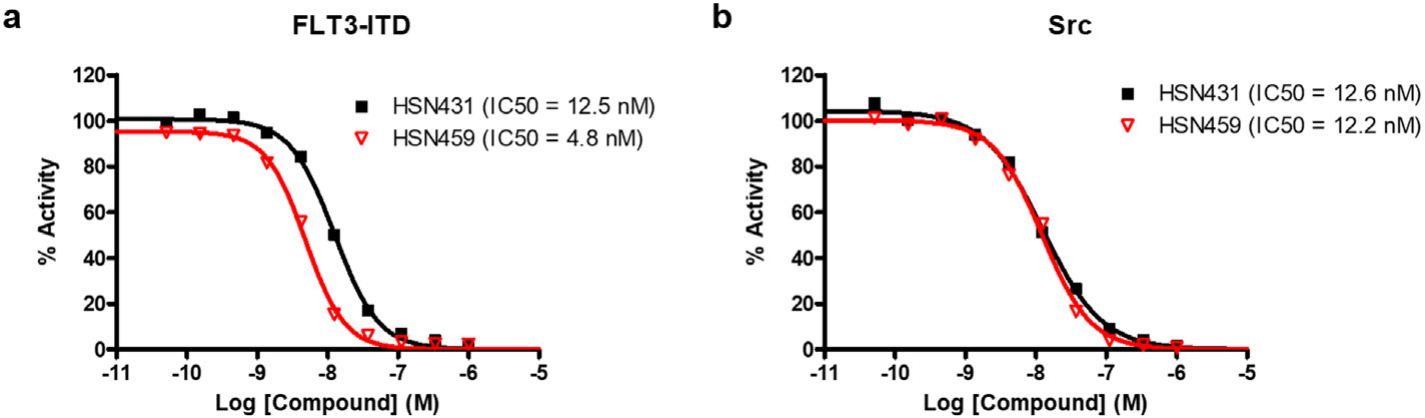
*In vitro* kinase inhibition activity of select compounds. Inhibition of (a) FLT3-ITD (100 nM) and (b) Src (0.6 nM) by HSN431 and HSN459. IC50 values of each compound are shown in parenthesis. ATP concentration was 100 μM. Substrates used and substrate concentrations are presented in Table S1. Each data point represents the mean ± SD of duplicate measurements.

To further probe the kinase inhibitory activity, we proceeded to determine the IC50 values of the compounds against FLT3-ITD and Src. Since almost all the compounds had excellent FLT3 and Src inhibition (Table S2), we chose representative compounds for isoquinolines (HSN431) and naphthyridines (HSN459). HSN431 was equipotent against both kinases (12.5 nM against FLT3-ITD and 12.6 nM against Src kinase). HSN459 had an IC50 of 4.8 nM against FLT3-ITD and 12.2 nM against Src (Fig. 3).

## 4 Compounds inhibit AML cells harboring FLT3 TKD mutations

Having established that the second-generation compounds were potent inhibitors of MV4–11 cell proliferation (most compounds inhibited MV4–11 cells with IC50 <10 nM), we proceeded to evaluate the activities of the compounds against drug-resistant AML cell lines. As previously stated, the majority of FLT3 inhibitors provide initial response in the clinic but none provide complete cure due to the emergence of resistance mechanisms. One of the major resistance mechanisms to FLT3 inhibitors is the emergence of a secondary mutation in the kinase domain [30,31]. Patients with double FLT3-ITD secondary TKD mutations have poorer prognosis than those with single FLT3-ITD or FLT3 TKD mutations. For example sorafenib, an FDA-approved drug for thyroid, liver and kidney cancers, potently binds to FLT3 and FLT3-ITD with *K*_*d*_ values of 13 nM and 95 nM respectively and has shown activity against AML in the clinic [32]. But sorafenib is a poor binder of FLT3-ITD (D835V) or FLT3-ITD (F691 L) with *K*_*d*_ values of 630 nM and 860 nM respectively and resistance to sorafenib due to secondary mutations in FLT3 has been documented [18]. Midostaurin and newer generation FLT3 inhibitors, such as crenolanib, which are type I inhibitors fare better in binding to FLT3-ITD (F691 or D835) secondary mutated kinases but as already mentioned midostaurin is not active as a single agent [11,12] and crenolanib suffers from pharmacokinetic issues [33] and requires multiple dosing regimens. The need for novel FLT3-targeted AML therapeutics that are active against the problematic secondary mutations can therefore not be overstated. Pleasingly, a handful of the compounds that showed excellent activities against FLT3-ITD-harboring MV4–11 cells, also showed very good activity (using the MTT assay) against the drug-resistant MOLM-14 cell lines that harbored F691 L and D835Y secondary mutations. To confirm that the compounds were killing the AML cell lines, rather than just inhibiting growth, we used trypan blue exclusion assay to count viable cells after incubating MV4–11 with one model compound, HSN431. The cell count indicated that viable MV4–11 cells decreased in presence of compound in dose-dependent manner (Fig. S1).

### 4.1 Active compounds possess *in vivo* efficacy against MV4–11 cells

**Fig. 4:**
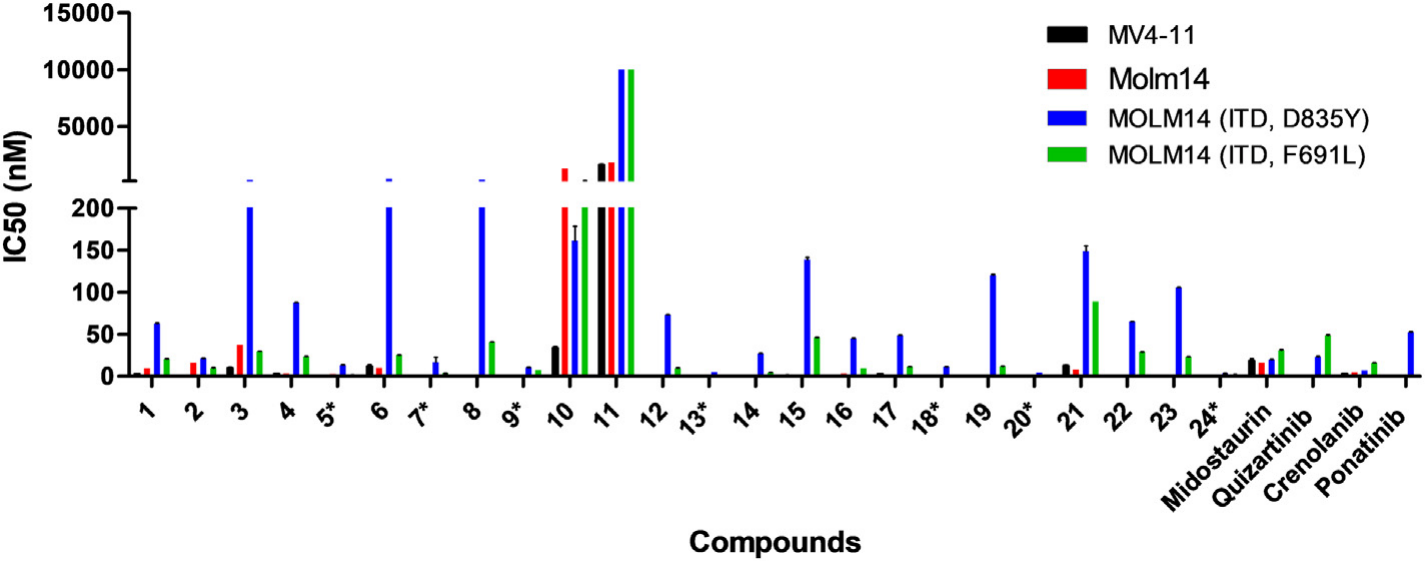
Anti-proliferative activity of second-generation compounds. The IC50 values against MV4–11 (black), MOLM-14 (red), MOLM-14 (ITD, D835Y) (blue) and MOLM-14 (ITD, F691 L) (green) cells were plotted for each compound. Compounds with 15 nM or less IC50 against all 4 cell lines are marked with *. Each bar represents the average of triplicates plotted using GraphPad Prism software (GraphPad, La Jolla, CA). (For interpretation of the references to colour in this figure legend, the reader is referred to the web version of this article.)

**Fig. 5:**
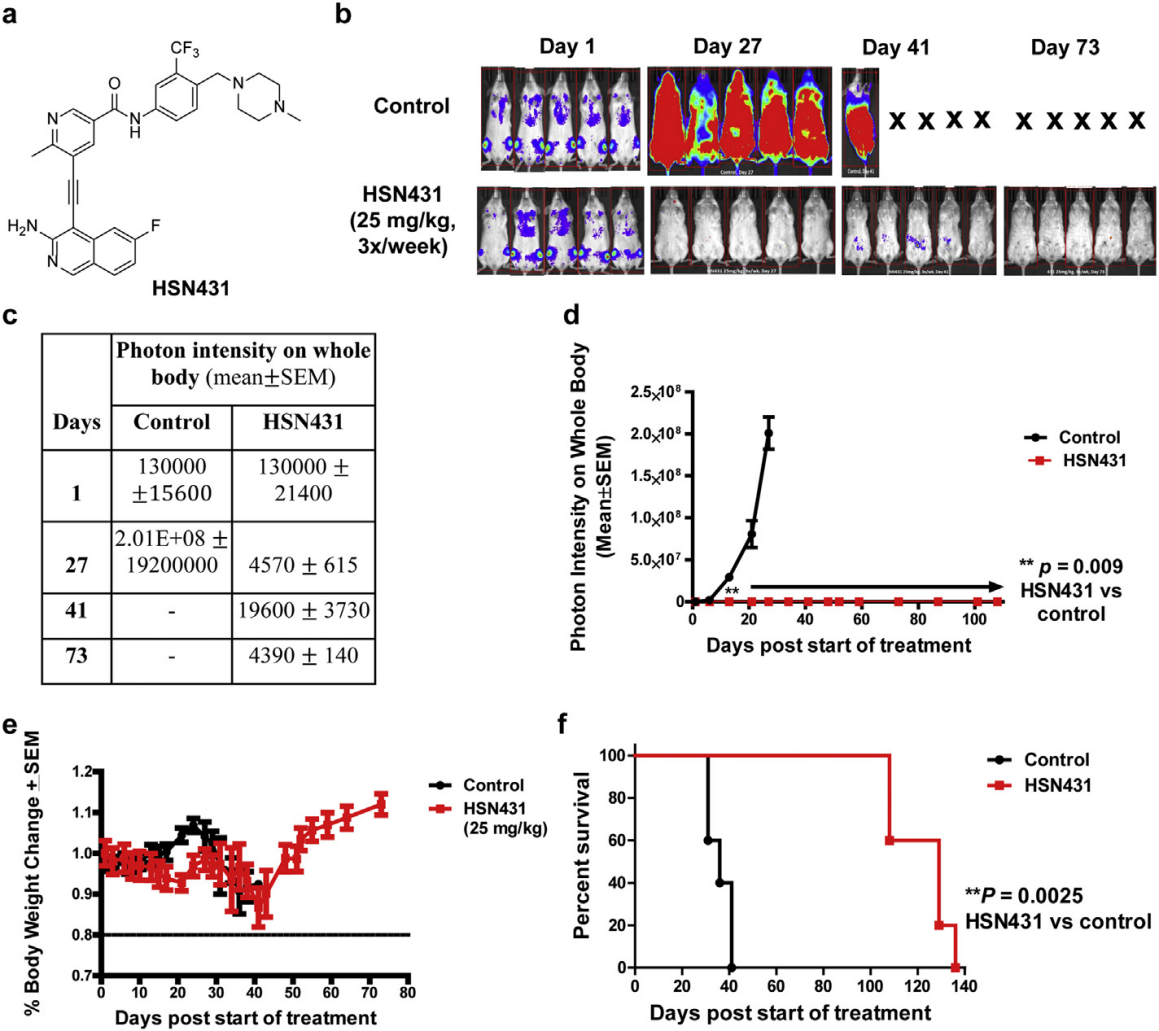
Evaluation of the *in vivo* efficacy of HSN431. (a) Structure HSN431. (b) The effect of HSN431 (25 mg/kg IP; 3×/week) treatment on mice (*n* = 5) implanted with FLT3-ITD harboring MV4–11 cells. (c) Table of photon intensity on whole body values for images shown in (b). (d) Quantification of leukemia burden in mice by measuring photon intensity (emanating from bioluminescent MV4–11 cells) on whole body of different treatment groups (control *versus* HSN431) over time. (e) Body weight measurements of control *versus* HSN431 treatment. (f) Survival traces for the mice treated with HSN431 and control until day 140. **P < .01.

**Fig. 6:**
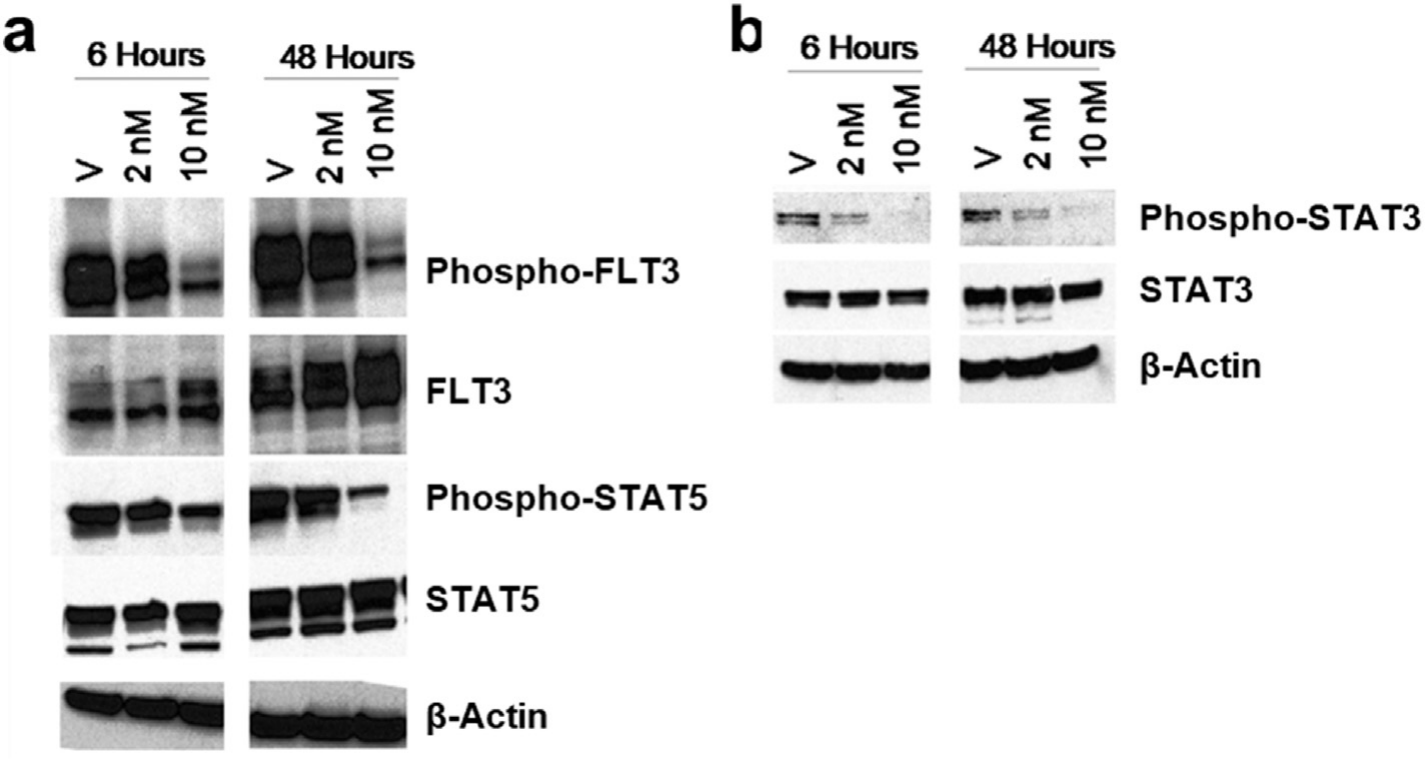
Evaluation of the effect of HSN431 on protein phosphorylation in MV4–11 cells. Cells were treated with 2 nM or 10 nM HSN431 for 6 h and 48 h and protein extracts were probed with primary antibodies of (a) phospho-FLT3, FLT3 phospho-STAT5 and STAT5 and (b) phospho-STAT3 and STAT3. Primary antibodies of indicated kinases or proteins were detected using anti-rabbit or anti-mouse secondary antibodies. Scanned images were analyzed using image J software. For full immunoblots, see Fig. S6.

**Fig. 7:**
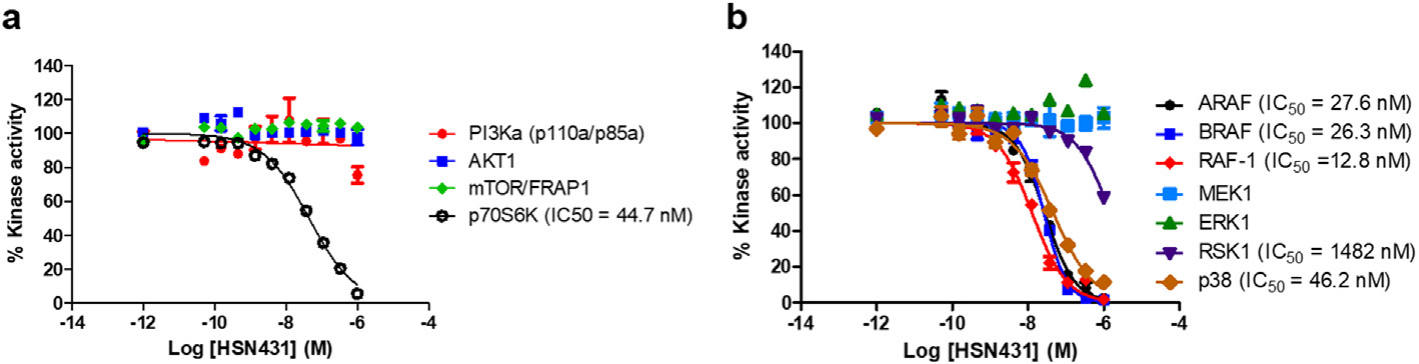
*In vitro* kinase inhibition by HSN431. The dose-response curves obtained by incubating increasing concentrations of HSN431 with kinases in the (a) PI3K/AKT/mTOR pathway and (b) MAPK pathway. ATP concentration was 100 μM. Refer to Table S1 for concentrations of kinases and corresponding substrates. Each data point represents the mean ± SD of duplicate measurements.

Compounds HSN431, HSN459, 7, 11, 15, 20 and 26, inhibited MOLM-14 (FLT3-ITD, D835Y) and MOLM-14 (FLT3-ITD, F691 L) with IC50 values that are 15 nM or lower (see Fig. 4 and Table 1). HSN459, HSN431 as well as compounds 5, 9, 13, 18 and 24, were impressive as they were more active against MV4–11 cells and drug-resistant MOLM-14 cell lines than even the FDA-approved FLT3 inhibitor, midostaurin, or most FLT3 compounds in clinical trials (crenolanib, quizartinib and ponatinib, see Fig. 4 and Table 1). The ultimate goal of this study was to identify a preclinical compound that could be translated into an AML therapeutic, so we proceeded to evaluate the compounds against MV4–11 cells in mice (Fig. 5, Fig. 6, Fig. 7 and S2–S5).

**Table 1:** Summary of anti-proliferative activities of select compounds.

**Compounds**	**IC50 (mean *±*SD, nM)**			
	**MV4–11**	**MOLM-14**	**MOLM-14 (ITD, D835Y)**	**MOLM-14 (ITD, F691** **L)**
**5 (HSN580)**	0.4±0.004	2.8±0.02	13.0±0.4	1.5±0.1
**7 (HSN431)**	0.5±0.01	0.5± 0.01	16.6±5.8	3.3±0.3
**9**	0.4±0.04	2.3± 0.1	10.4±0.3	6.9±0.05
**13**	0.4±0.006	1.4± 0.03	4.7±0.03	1.1±0.04
**18**	0.2±0.001	0.2±0.002	11.1±0.08	1.1±0.04
**20 (HSN459)**	0.3±0.004	0.2±0.002	4.0±0.02	0.9±0.004
**24**	0.2±0.009	0.2± 0.01	3.0±0.3	1.8±0.08
**Midostaurin**	18.5±2.4	16.1±0.02	19.6±0.3	30.7±0.7
**Quizartinib**	0.4±0.02	0.5±0.02	23.0±0.5	48.5±0.6
**Crenolanib**	3.1±0.06	4.5±0.8	6.6±0.03	15.6±0.4
**Ponatinib**	0.1±0.001	0.5± 0.1	52.6±0.3	6.8±0.03

The compounds that displayed potent activities against the AML cell lines *in vitro* (HSN286, HSN334, HSN352, HSN356, HSN431, HSN459, HSN461, HSN580, HSN581 and HSN620) were advanced for *in vivo* tolerability and efficacy studies on six-week-old NSG or NRG mice (see Figs. 5 & S2–S5). Female mice were dosed daily for 5 days *via* intraperitoneal injection and then monitored for an additional week for weight loss, a sign of toxicity in mice. It was found that when administered intraperitoneally, most of the compounds were tolerated at 25 mg/kg (no or transient weight loss <20% body weight). To evaluate the anti-leukemic efficacy *in vivo*, six-week-old female NSG or NRG mice were injected intravenously with FLT3-ITD expressing acute myeloid leukemia cells (MV4–11), expressing luciferase. Ten days after the initial MV4–11 injection, most of the mice showed some leukemia burden. Dosing (i.p) with the compounds was then started. The mice were dosed with 25 mg/kg of the compounds three times a week (MWF). Apart from HSN286, most of the tested compounds showed >99% reduction in leukemia burden after a few weeks (see Figs. 5 & S2–S5). HSN431, HSN580 and HSN459 were notable and it appeared that these compounds had cleared >99% of the leukemia in mice after few days. At 25 mg/Kg, the mice in the HSN459 group lost weight after one week so the dosing for this group was reduced to 10 mg/kg, which was tolerable (see Fig. S5).

Since HSN431 could be dosed for several weeks without any adverse events and all five mice were still alive after day 73 (see Fig. 5; all the control mice had died by day 43), HSN431 was chosen as a model lead candidate and characterized further (Western analysis, cell cycle and apoptosis analysis).

In the wildtype FLT3 signaling, activation of FLT3 occurs through binding to FLT3 ligand and subsequent phosphorylation of its TKD [34]. This causes the phosphorylation and activation of several downstream effector proteins *via* signaling pathways like the phosphatidylinositol-3-kinase (PI3K) - protein kinase B (Akt) and mitogen-activated protein kinase (MAPK) pathways [34]. These signaling pathways interact with each other and ultimately result in cell proliferation and survival [34]. Constitutive activation of FLT3 through mutations leads to constitutively active downstream effectors and as such affords an enhanced cell survival and proliferation. We used Western blot analysis to investigate the effect of HSN431 on the phosphorylation of FLT3 and downstream effectors in FLT3-ITD expressing MV4–11 cells (Figs. 6 & S6). In agreement with our *in vitro* kinase results (Fig. 3 & Table S2), we observed an inhibition of the phosphorylation of FLT3 (Fig. 6). Inhibition of FLT3 phosphorylation was also observed in MOLM-14 (ITD, D835Y) cells (Fig. S7). In MV4–11 cells, inhibition of the phosphorylation of Src was also observed (Fig. S8). Intriguingly, we found that after 48 h, the effect of HSN431 on Src phosphorylation was lower at 10 nM compared to 2 nM compound (Fig. S8). Although this was reproducible, the molecular basis is unclear and will be elucidated in future publications. We however, postulate that at higher concentrations, the compound could affect other pathways that modulate Src phosphorylation. Constitutive activation of Signal Transducer and Activator of Transcription 3 and 5 (STAT3 and STAT5) have been observed as a result of Src and FLT3 activation respectively [35,36]. Consistent with the inhibition of Src and FLT3 phosphorylation, STAT3 and STAT5 phosphorylation were inhibited (Figs. 6, S6 & S8). From the Western blot analysis, we also observed decreased phosphorylation of both Akt and ribosomal protein S6 kinase beta-1 (p70S6K) (Fig. S8), kinases in the PI3K-Akt-mTOR pathway that is activated in FLT3 signaling [34]. Phosphorylation and activation of Akt leads to the activation of mammalian target of rapamycin (mTOR) which in turn activates p70S6K *via* phosphorylation [34]. Consequently, inhibition of Akt phosphorylation would lead to decreased phosphorylation of p70S6K as observed (Fig. S8). The activation of p70S6K leads to increased protein translation, which is important for cell survival [34,37,38]. Interestingly, from cell-free *in vitro* kinase assays, we found that HSN431 inhibited the kinase activity of p70S6K with an IC50 of 44.7 nM but not the upstream kinases PI3K, Akt and mTOR (Fig. 7). Hence inhibition of FLT3 kinase activity (Fig. 3) may be responsible for the observed decrease in Akt and p70S6K phosphorylation.

The mitogen-activated protein kinase (MAPK) pathway has been demonstrated to be involved in leukemogenesis due to the activation of the Ras-Raf-MEK-extracellular signal-regulated kinase (ERK) cascade [39]. Down-regulation of this cascade results in cell cycle arrest and induces apoptosis [40,41]. Others have shown that the phosphorylation of p38 MAPK was enhanced in MV4–11 cells and p38 activity has been implicated in leukemogenesis [42,43]. We observed inhibition of the phosphorylation of ERK, p38 and a modest inhibition of c-Myc which is downstream of p38 by HSN431 (Fig. S8). For inhibition of ERK phosphorylation, we found that HSN431 appeared to inhibit ERK phosphorylation better after 6 h than after 48 h (Fig. S8). Time-dependent increase in ERK phosphorylation following initial decrease has been observed by others in MV4–11 and MOLM-14 cell lines [44]. Further analysis by kinase inhibition assays revealed that HSN431 inhibited the kinase activity of A-Raf (IC50 = 27.6 nM), B-Raf (IC50 = 26.3 nM) and Raf-1 or c-Raf (IC50 = 12.8 nM) but not the downstream effectors MEK1, ERK1 and RSK1 (Fig. 7). The observation that HSN431 inhibits the Raf kinase activity, which is required for the activation of ERK, potentially explains the observed decrease in cellular ERK phosphorylation.

### 4.2 HSN431 induces G1 cell cycle arrest and apoptosis

**Fig. 8:**
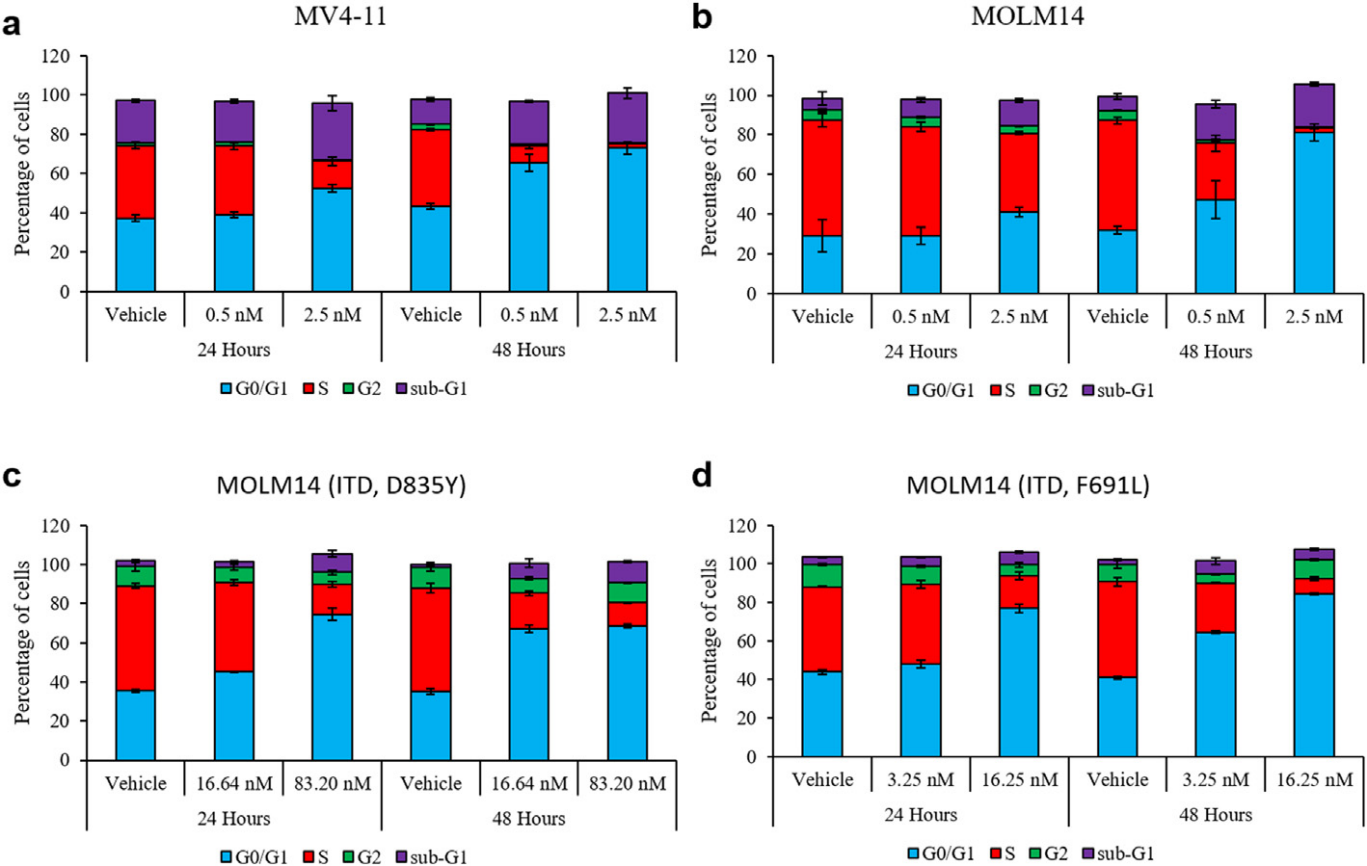
The effect of HSN431 on cell cycle distribution. DMSO (vehicle) or HSN431 at the indicated concentrations was incubated with (a) MV4–11, (b) MOLM-14, (c) MOLM-14 (ITD, D835Y) and (d) MOLM-14 (ITD, F691 L) cells for 24 h and 48 h. Cells were harvested by centrifugation, washed, fixed in 70% ethanol and stained with propidium iodide (PI) before being analyzed by flow cytometry. For each stacked bar, the distribution of cells in the G1 phase (blue), S phase (red), G2 phase (green) and sub-G1 (magenta) have been indicated by their percentages. Samples were analyzed in triplicates on a FACSCanto II flow cytometer (BD Bioscience, San Jose, CA, USA) and data were analyzed with FlowJo software (Flowjo, LLC, Ashland, OR, USA). Data represent the mean ± SD of triplicate measurements. (For interpretation of the references to colour in this figure legend, the reader is referred to the web version of this article.)

**Fig. 9:**
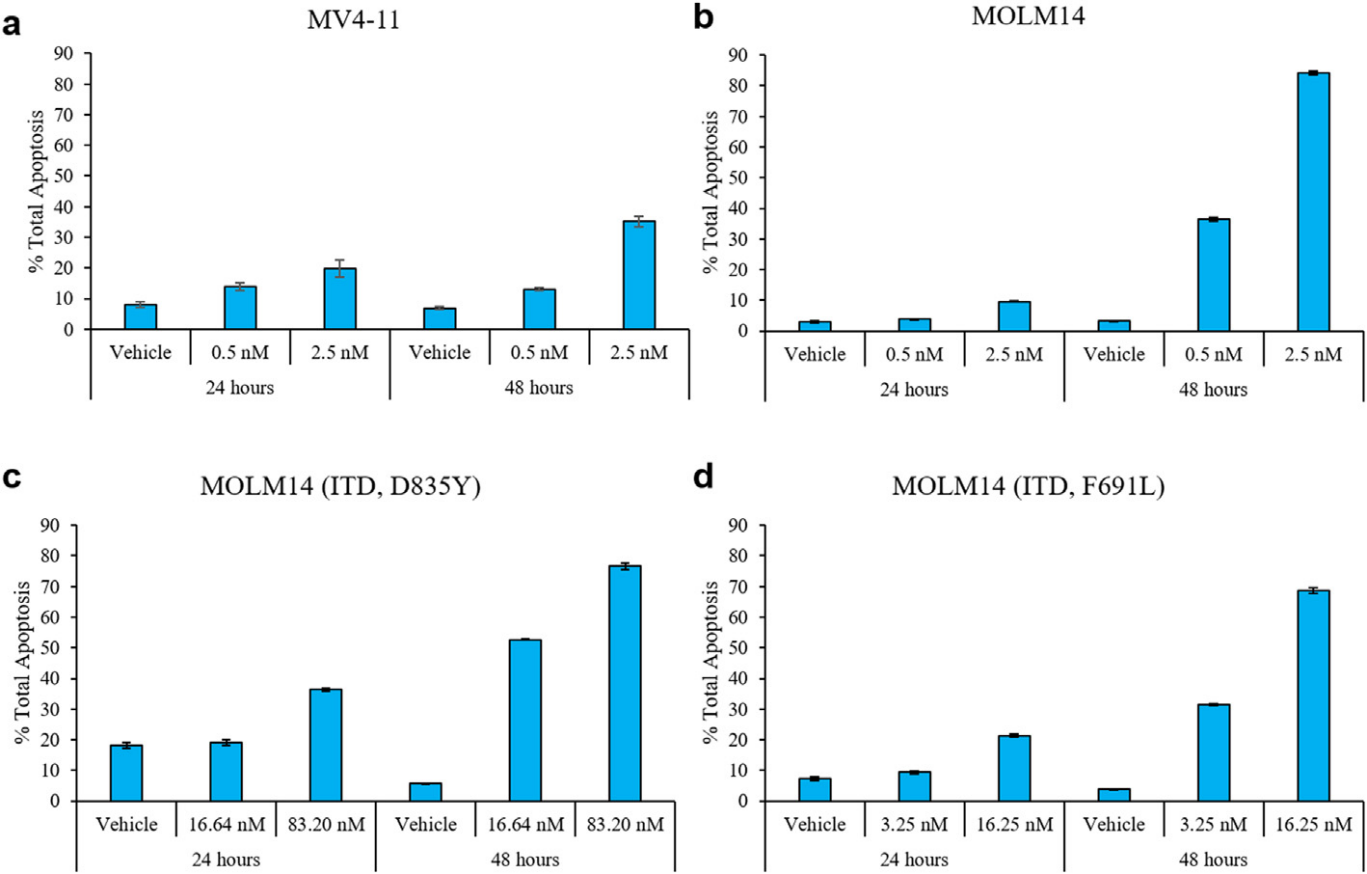
HSN431 induces apoptosis in AML cells. Flow cytometry analysis of the total apoptosis of (a) MV4–11, (b) MOLM-14, c) MOLM-14(ITD, D835Y) and d) MOLM-14 (ITD, F691 L) cells treated with DMSO (vehicle) or the indicated concentrations of HSN431 for 24 and 48 h. At the indicated time-points, cells were harvested, washed and stained with FITC Annexin V and PI and analyzed by flow cytometry on a FACSCanto II flow cytometer. Total apoptosis (%) was defined as the sum of percentages of early and late apoptotic cells. Experiment was performed in triplicates and presented as mean ± SD.

The effect of HSN431 on cell cycle in the AML cell lines was evaluated. From the cell cycle analysis, an increase in the population of cells in the G0/G1 phase was observed when the AML cell lines tested were treated with HSN431. The accumulation of G0/G1 phase cells was observed to be both dose- and time-dependent. Also, corresponding decreases in the distribution of cells in the S phase (Figs. 8 and S9) were observed. A general decrease in the distribution of cells in the G2 phase was also observed. Additionally, we also observed an accumulation of cells in the sub-G1 phase across the tested AML cell lines. A further analysis of the antileukemic effect of HSN431 revealed that the compound induced apoptosis in the AML cell lines. Again, we observed both dose- and time-dependent increases in total apoptosis (Figs. 9 and S10).

## 5 Discussion

Several reports have documented that the inhibition of FLT3 [1,45] or Src kinase [46] is an attractive strategy to inhibit the proliferation of AML. Whereas FLT3 plays a key role in leukemogenesis in about 30% of AML patients [6,7], Src-family kinases, which are downstream of FLT3, are over-expressed in AML and collaborate with FLT3 to facilitate AML progression [46]. In addition to Src, HCK and Lyn (both are Src kinase family members) are inhibited by the reported aminoisoquinolines [24]. HCK is known to be expressed in human primary leukemic stem cells and not as much in human normal hematopoietic stem cells. Lyn is known to play a key role in the progression of AML [47]. Indeed dasatinib, a Src-family inhibitor, which is an FDA approved drug for CML has been shown to be effective against AML [46]. We had earlier reported that aminoisoquinoline benzamides are dual FLT3-Src-family kinase inhibitors with potent activities against FLT3-ITD harboring AML cell lines MV4–11 and MOLM-14 [24]. The first-generation compounds (such as HSN286, HSN334 and HSN356) (Fig. 1) were however not drug-like (high LogP values for example) and were poorly active against AML with secondary FLT3 mutations. However, the second-generation alkynyl aminoisoquinoline and alkynyl aminonaphthyridine compounds (Fig. 2) had reduced LogP values and possessed potent activity against AML cell lines harboring secondary FLT3 mutations (Fig. 4).

AML patients who harbor FLT3-ITD mutations have worst prognosis [48]. Most FLT3 TKIs in clinical trials including crenolanib, quizartinib and gilteritinib as well as midostaurin, the first FDA approved FLT3 TKI, are effective against FLT3-ITD mutations [11,33,49,50]. It has emerged that, the activation loop mutation D835Y/V as well as the gatekeeper mutation F691 L contribute significantly to resistance to FLT3 TKIs, consequently leading to AML relapse [18,25,51]. The ability of a FLT3 TKI to inhibit these mutant forms could translate into better clinical outcomes. HSN431, HSN459, and compound 24 among others, exhibited similar or better anti-leukemic activity against drug-resistant AML cells including MOLM-14 cells harboring double FLT3-ITD-D835Y and FLT3-ITD-F691 L mutations compared to midostaurin, crenolanib, quizartinib and ponatinib.

In an *in vivo* mouse xenograft model, using MV4–11 model that was previously used to evaluate crenolanib [33], HSN431 at 25 mg/kg and dosed three times per week drastically reduced the whole-body photon count due to the bioluminescence of MV4–11-luc cells, compared to control. Furthermore, HSN431 treatment improved mice survival. By day 40, all the control mice had died whereas 100% of HSN431-treated mice were still alive (Fig. 5).

HSN431 is a multitargeted kinase inhibitor and it is likely that the potent activity *in vivo* against AML is due to the ability to not only inhibit FLT3 but some of the downstream targets. In fact, most kinase inhibitors, including even ones that the investigators claimed were selective agents, have now been shown to target multiple targets [52]. Plausibly the clinical successes of the many FDA-approved TKIs, which are multitargeting kinase inhibitors, derive from their ability to inhibit the signaling pathway that lead to tumor/cancer growth (*i.e.* cancer driver kinase and downstream “collaborating” kinases) as well as kinases that regulate the tumor microenvironment [53] and/or immunokinases, which either activate cytotoxic T-cells and/or inhibit suppressor immune cells [54]. A limitation of multitargeting kinase inhibitors is the issue of dose-limiting toxicities, due to the inhibition of kinases that are also essential for normal physiological function. Irrespective of mode(s) of action, increasing survival of patients whiles not drastically impacting quality of life (due to adverse events) should be the main criteria for a drug selection. In this regard, we believe that HSN431 has a potential to be translated in the clinic since it could be dosed to mice for several weeks without any obvious signs of toxicity, such as weight loss or animal discomfort, and importantly drastically improved survival of mice injected with AML cells (Fig. 5).

In summary, we have developed alkynyl aminoisoquinoline and alkynyl naphthyridine compounds with potent *in vitro* FLT3-Src kinase inhibition. The compounds demonstrated potent activities against TKI-resistant FLT3-ITD cell lines and were efficacious in *in vivo* mouse AML studies. HSN431 and analogs thereof could be developed further as anti-AML compounds.

## Funding sources

Purdue University, Purdue Institute for Drug Discovery (PIDD), Purdue University Center for Cancer Research and Elks Foundation provided funding. NMR and MS data were acquired by the NMR and MS facilities supported by NIH P30 CA023168.

Herman Sintim (corresponding author) had full access to all the data in the study and had final responsibility for the decision to submit for publication. The funders had no roles in study design, data collection, analysis, interpretation or writing the manuscript.

## Disclosure statement

Dr. Lapidus is a co-founder of KinaRx LLC, a start-up company interested in developing therapies for malignant neoplastic diseases. Dr. Sintim reports grants from Elks Foundation, grants from NIH (P30 Center Grant), during the conduct of the study; In addition, Dr. Sintim has a patent PCT/US17/46843 pending and Dr. Sintim is a co-founder of KinaRx LLC, a start-up company interested in developing therapies for malignant neoplastic diseases.

## Author contributions

H.O.S. designed overall study, managed overall study and secured funding for study. R.G.L. and B.D.E. designed animal efficacy studies. R.G.L. supervised work done by E.Y.C., E.T.C. and B.A.C-C. B.D.E. supervised work done by S.E.T-A.

H.O.S. and C·O-T. wrote the manuscript. E.L., R.G.L. and B.D.E. edited the manuscript. N.N. synthesized compounds. C·O-T., E.L., B.A.C-C. performed viability studies on AML cell lines. C·O-T., M.W., B.A.C-C. performed Western assays. C·O-T. and B.A.C-C. performed apoptosis assays. B.A.C-C. performed cell cycle experiments. E.Y.C., E.T.C. and S.E.T-A. performed *in-vivo* efficacy studies. H.O.S., C·O-T., R.G.L. and B.D.E. analyzed and interpreted data.

## Appendix A Supplementary data

Supplementary material Image 1
