# Supplementary material for: Amino alkynylisoquinoline and alkynylnaphthyridine compounds potently inhibit acute myeloid leukemia proliferation in mice
Source: eBioMedicine. 2019 Jan 25;40:231–9. doi: 10.1016/j.ebiom.2019.01.012 (PMC6413339; doi:10.1016/j.ebiom.2019.01.012)

Supplementary Information

**Amino alkynylisoquinoline and alkynylnaphthyridine compounds potently inhibit acute myeloid leukemia proliferation in mice.**

N Naganna^1,2,§^, Clement Opoku-Temeng^1,2,4,§^, Eun Yong Choi,^5^ Elizabeth Larocque^1,2^, Elizabeth T. Chang^5^, Brandon A. Carter-Cooper^5^, Modi Wang^1,2^, Sandra E. Torregrosa-Allen^3^, Bennett D. Elzey^3^, Rena G. Lapidus^5^ and Herman O. Sintim^1,2,3,6^*

^1^Purdue Institute for Drug Discovery, West Lafayette IN 47907, USA

^2^Department of Chemistry, Purdue University, West Lafayette IN 47907, USA

^3^Purdue University Center for Cancer Research, Purdue University, West Lafayette IN 47907, USA

^4^Graduate Program in Biochemistry, University of Maryland, College Park, MD 20742, USA

^5^University of Maryland School of Medicine, Baltimore, MD

^6^Lead Contact

^§Equal contribution^

*Correspondence: [hsintim@purdue.edu](mailto:hsintim@purdue.edu)

- - - 1. **Supplementary data**

**Table S1.** Summary of kinase reactions performed at Reaction Biology Corporation

| **Kinases** | **Kinase Vendor** | **Kinase Catalog #** | **Kinase in Rxn** | **Substrate** | **Substrate Vendor** | **Substrate Catalog #** | **Substrate in Rxn** |
| --- | --- | --- | --- | --- | --- | --- | --- |
| **FLT3** | Invitrogen | PV3182 | 15 nM | ABLtide | GenScript | 94851_7 | 20 µM |
| **c-Src** | Invitrogen | PR4336E | 0.6 nM | pEY | Sigma | P7244 | 0.2 mg/mL |
| **ERK1** | Invitrogen | PR5254B | 20 nM | MBP | Active Motif | 102641 | 20 µM |
| **mTOR/FRAP1** | Invitrogen | PV4754 | 300 nM | 4EBP1/E1F4EBP1 + Mn^2+^ | Reaction Biology | SUB-11-444 | 10 µM |
| **BRAF** | BPS | 40005 | 10 nM | MEK1 (K97R) | Reaction Biology | RBC-MEK1 (K97R) | 5 µM |
| **RAF1** | Invitrogen | PV3805 | 4 nM | MEK1 (K97R) | Reaction Biology | RBC-MEK1 (K97R) | 5 µM |
| **ARAF** | BPS | 40010 | 200 nM | MEK1 (K97R) | Reaction Biology | RBC-MEK1 (K97R) | 5 µM |
| **P38a/MAPK14** | Invitrogen | PV3304 | 20 nM | MBP | Active Motif | 102641 | 20 µM |
| **AKT1** | Invitrogen | PR3878D | 8 nM | Crosstide | Enzo | BML-P149 | 20 µM |
| **MEK1** | Invitrogen | PV3303 | 90 nM | ERK2 (K52R) | Reaction Biology | RBC-ERK2 (K52R) | 5 µM |
| **RSK1** | Invitrogen | PR6812A | 2 nM | Glycogen Synthase-derived peptide | GenScript | 56875_2 | 20 µM |
| **p70S6K/RPS6KB1** | Invitrogen | PV3815 | 3 nM | S6K/Rsk2 peptide 2 | GenScript | U2208BJ140_1 | 20 µM |
| **PI3Ka (p110a/p85a)** | Invitrogen | PR8581C | 4.5 µM | PI(4,5)P2:PS | SignalChem | P429-59 | 10 µM |

**Table S2**. Summary of the enzymatic and anti-proliferative activity of second-generation compounds

| **Compound** | **cLogP^a^** | **LogD (7.4)^b^** | **%FLT3 inhibition^c^** | **%Src**  **inhibition^c^** | **% viability inhibition MV4-11^d^**  **Molm13-res^e^**  **Molm14 (ITD, D835Y)^f^**  **Molm14 (ITD, F691L)^g^** |
| --- | --- | --- | --- | --- | --- |
| **1** | 3.7 | 4.5 | 96 | 99 | 2.6^d^  59.1^e^  62.7^f^  20.4^g^ |
| **2** | 3.7 | 4.5 | 99 | 99 | 0.5^d^  9.7^e^  21.1^f^  9.6^g^ |
| **3** | 4.2 | 4.6 | 81 | 99 | 10.1^d^  116.4^e^  286.2^f^  29.3^g^ |
| **4**  **(HSN352)** | 5.02 | 5.5 | 93 | 100 | 3.1^d^  125^e^  87.7^f^  23.4^g^ |
| **5** | 3.8 | 3.9 | 99 | 100 | 0.4^d^  6.9^e^  5.9^f^  1.5^g^ |
| **6** | 4.2 | 5.6 | 90 | 99 | 12.0^d^  39.8^e^  406.5^f^  25.1^g^ |
| **7**  **(HSN431)** | 2.9 | 4.0 | 97 | 98 | 0.8^d^  8.2^e^  11.1^f^  6.3^g^ |
| **8**  **(HSN286)** | 5.7 | 6.1 | 97 | 99 | 0.5^d^  24.2^e^  318.2^f^  40.7^g^ |
| **9** | 4.4 | 4.5 | 98 | 98 | 0.4^d^  4.4^e^  10.4^f^  6.9^g^ |
| **10** | 4.9 | 5.3 | 75 | 93 | 33.9^d^  462.9^e^  161.1^f^  225.2^g^ |
| **11** | 5 | 5.6 | 43 | 16 | 1643^d^  843^e^  >10000^f^  >10000^g^ |
| **12** | 5.4 | 6.0 | 98 | 100 | 0.5^d^  9.4^e^  72.8^f^  9.7^g^ |
| **13** | 4.2 | 4.4 | 100 | 98 | 0.4^d^  0.3^e^  4.7^f^  1.1^g^ |
| **14** | 3.6 | 3.7 | 98 | 99 | 0.9^d^  6.8^e^  27.1^f^  4.3^g^ |
| **15**  **(HSN334)** | 4.9 | 5.5 | 100 | 97 | 1.4^d^  92.4^e^  138.8^f^  45.9^g^ |
| **16 (HSN461)** | 3.6 | 3.9 | 98 | 100 | 0.3^d^  1.8^e^  44.9^f^  9.5^g^ |
| **17 (HSN600)** | 4 | 5.6 | 98 | 98 | 2.8^d^  16.5^e^  48.7^f^  11.3 |
| **18** | 3.8 | 3.7 | 97 | 98 | 0.2^d^  3.2^e^  11.1^f^  1.1 |
| **19**  **(HSN356)** | 4 | 4.6 | 99 | 98 | 0.4^d^  67.8^e^  120.0^f^  11.2^g^ |
| **20**  **(HSN459)** | 2.7 | 3.0 | 97 | 100 | 0.1^d^  2.6^e^  4.1^f^  0.93^g^ |
| **21** | 3.5 | 3.8 | 80 | 20 | 12.7^d^  420.6^e^  149.0^f^  88.8^g^ |
| **22** | 5.2 | 5.7 | 98 | 100 | 0.2^d^  72.6^e^  65.0^f^  28.7^g^ |
| **23** | 4.3 | 4.9 | 99 | 99 | 0.9^d^  62.8^e^  105.6^f^  23.0^g^ |
| **24** | 3.1 | 3.3 | 100 | 98 | 0.2^d^  6.4^e^  3.4^f^  1.8^g^ |
| **Midostaurin** | 4.3 | 5.4 | 100 | ND | 18.5^d^  16.5^e^  19.6^f^  30.1^g^ |
| **Quizartinib** | 5.9 | 5.1 | 98 | ND | 0.4^d^  -^e^  23.0^f^  48.5^g^ |
| **Crenolanib** | 3.4 | 0.8 | 99 | ND | 3.1^d^  9.8^e^  6.6^f^  15.6^g^ |
| **Ponatinib** | 4.2 | 4.5 | 98 | ND | 0.1^d^  24.1^e^  52.6^f^  6.8^g^ |

^a^Calculated using online software at http://www.molinspiration.com

^b^LogD at pH 7.4 using online software at https://chemicalize.com

^c^Concentration of inhibitor = 500 nM. Values are average of duplicate.

ND represents not determined

**Figure S1.** **HSN431** affects MV4-11 cell viability. MV4-11 cells were incubated with increasing concentrations of **HSN431** for 72 h. The viable cells were then counted using trypan blue exclusion on the Countess automated cell counter (Life Technologies, Carlsbad, CA). Data represent mean ± SD of triplicates. Statistically significant difference determined by t test statistical analyses is indicated by * p≤0.05, **p≤0.01 and *** p≤0.001. Data was graphed using GraphPad Prism software (GraphPad, La Jolla, CA).


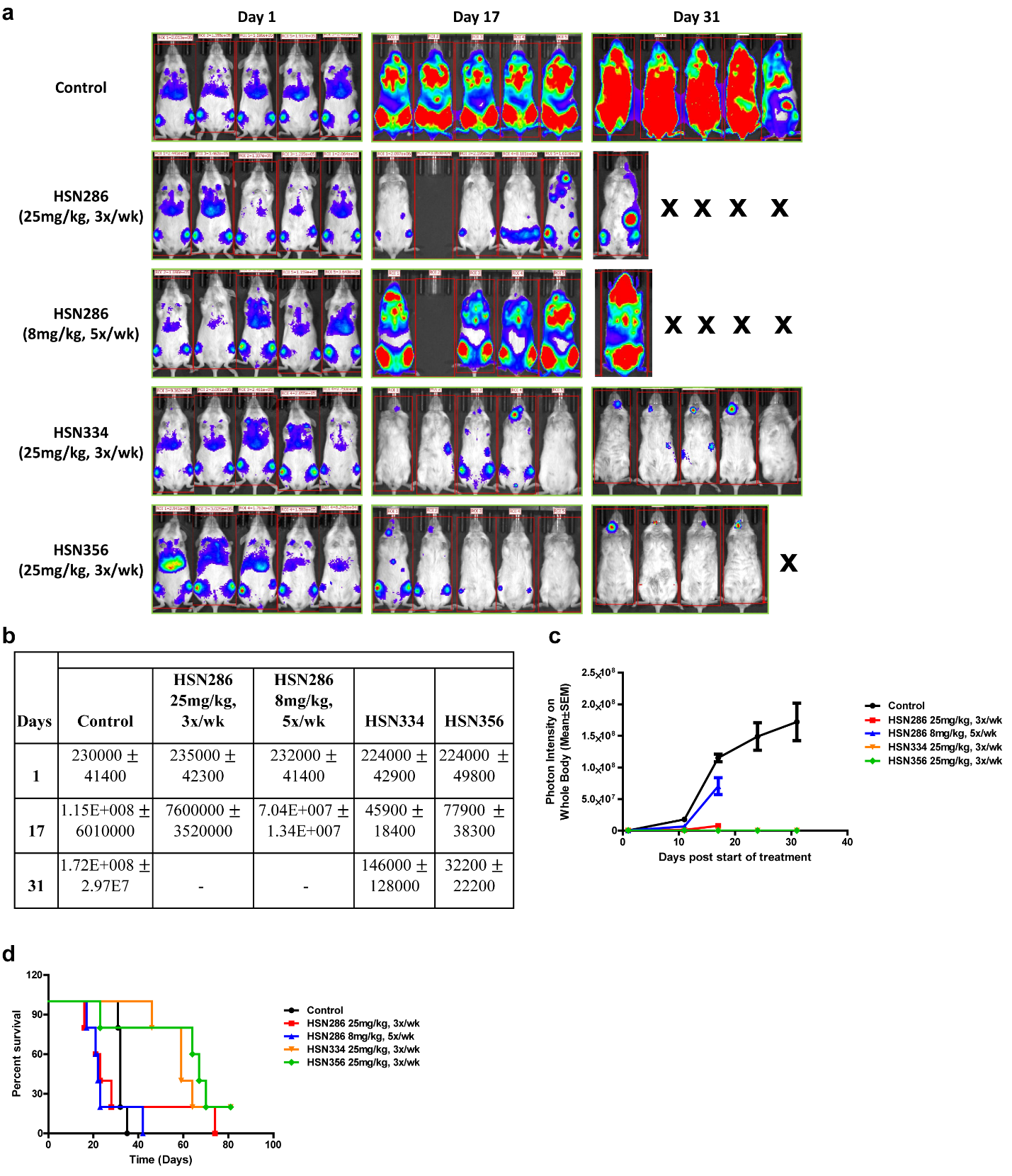


**Figure S2**. Evaluation of the *in vivo* efficacy of HSN286, HSN334 and HSN356. A) Treated mice were monitored by luciferase imaging. B) Table of photon intensity on whole body values for images shown in A. C) Qualitative measurement of photons produced by luminescent MV4-11 cells over time. D) Survival trace for treated and untreated control mice. Each data point represents the mean$\pm$SEM of all 5 mice from each group.


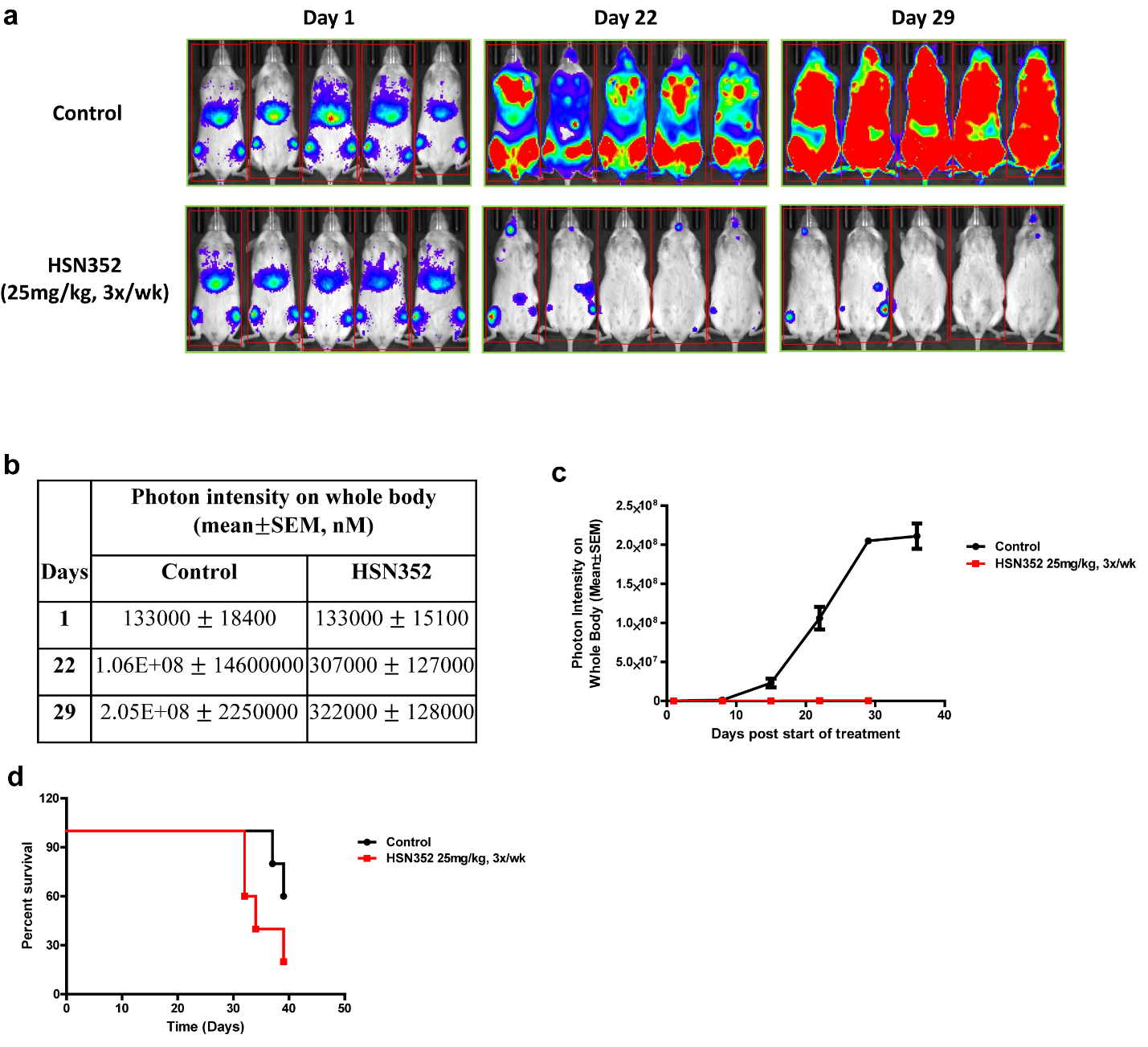


**Figure S3**. Evaluation of the *in vivo* efficacy of HSN352. A) Treated mice were monitored by luciferase imaging. B) Table of photon intensity on whole body values for images shown in A. C) Qualitative measurement of photons produced by luminescent MV4-11 cells over time. D) Survival trace for treated and untreated control mice. Each data point represents the mean$\pm$SEM of all 5 mice from each group.


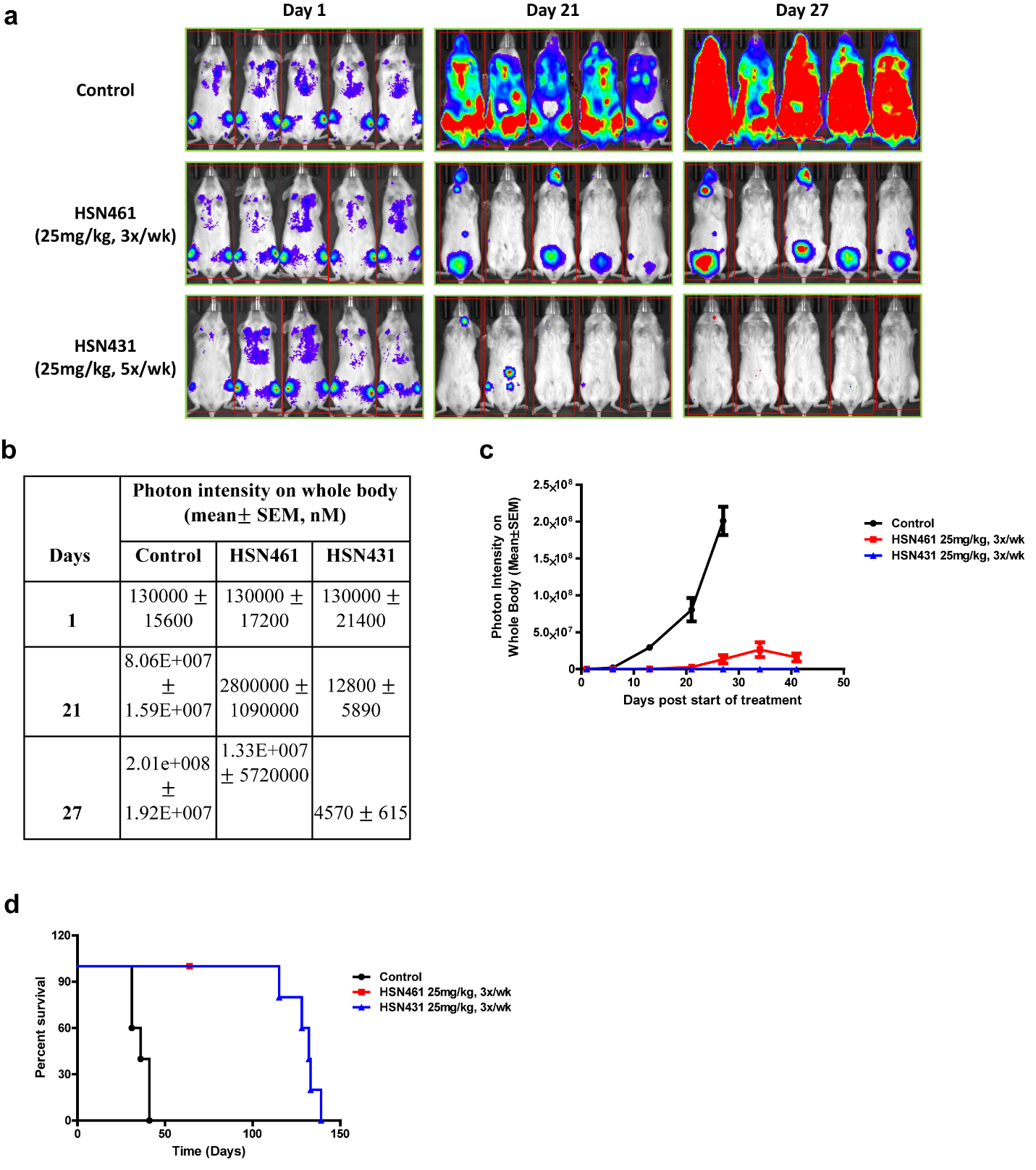


**Figure S4**. Evaluation of the *in vivo* efficacy of HSN461 and HSN431. A) Treated mice were monitored by luciferase imaging. B) Table of photon intensity on whole body values for images shown in A. C) Qualitative measurement of photons produced by luminescent MV4-11 cells over time. D) Survival trace for treated and untreated control mice. Each data point represents the mean$\pm$SEM of all 5 mice from each group.

**
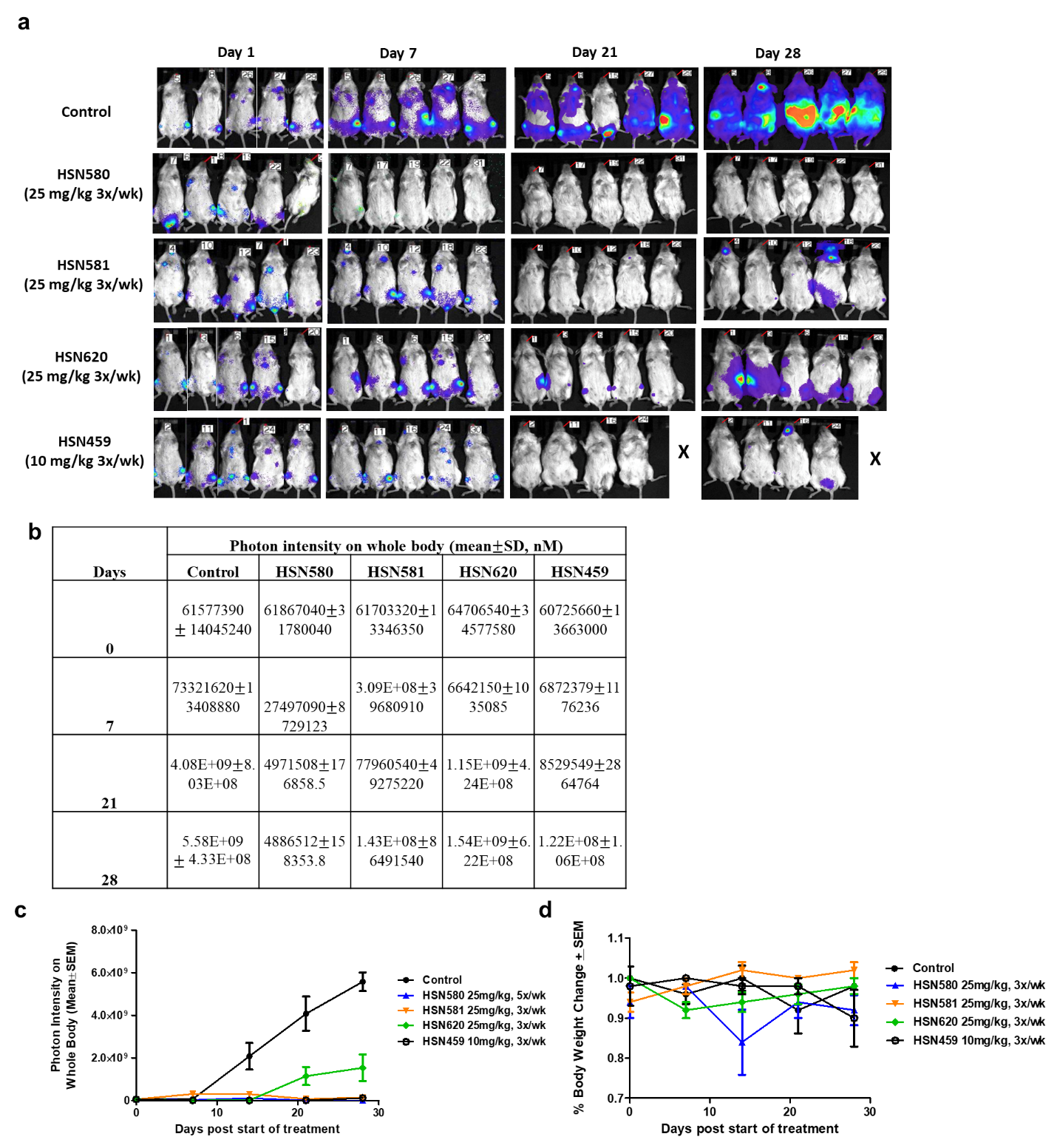
**

**Figure S5**. Evaluation of the *in vivo* efficacy of HSN580, HSN581, HSN620 and HSN459. A) Treated mice (NRG mice) were monitored by luciferase imaging. B) Table of photon intensity on whole body values for images shown in A. C) Qualitative measurement of photons produced by luminescent MV4-11 cells over time. D) Survival trace for treated and untreated control mice. Each data point represents the mean$\pm$SEM of all 5 mice from each group.


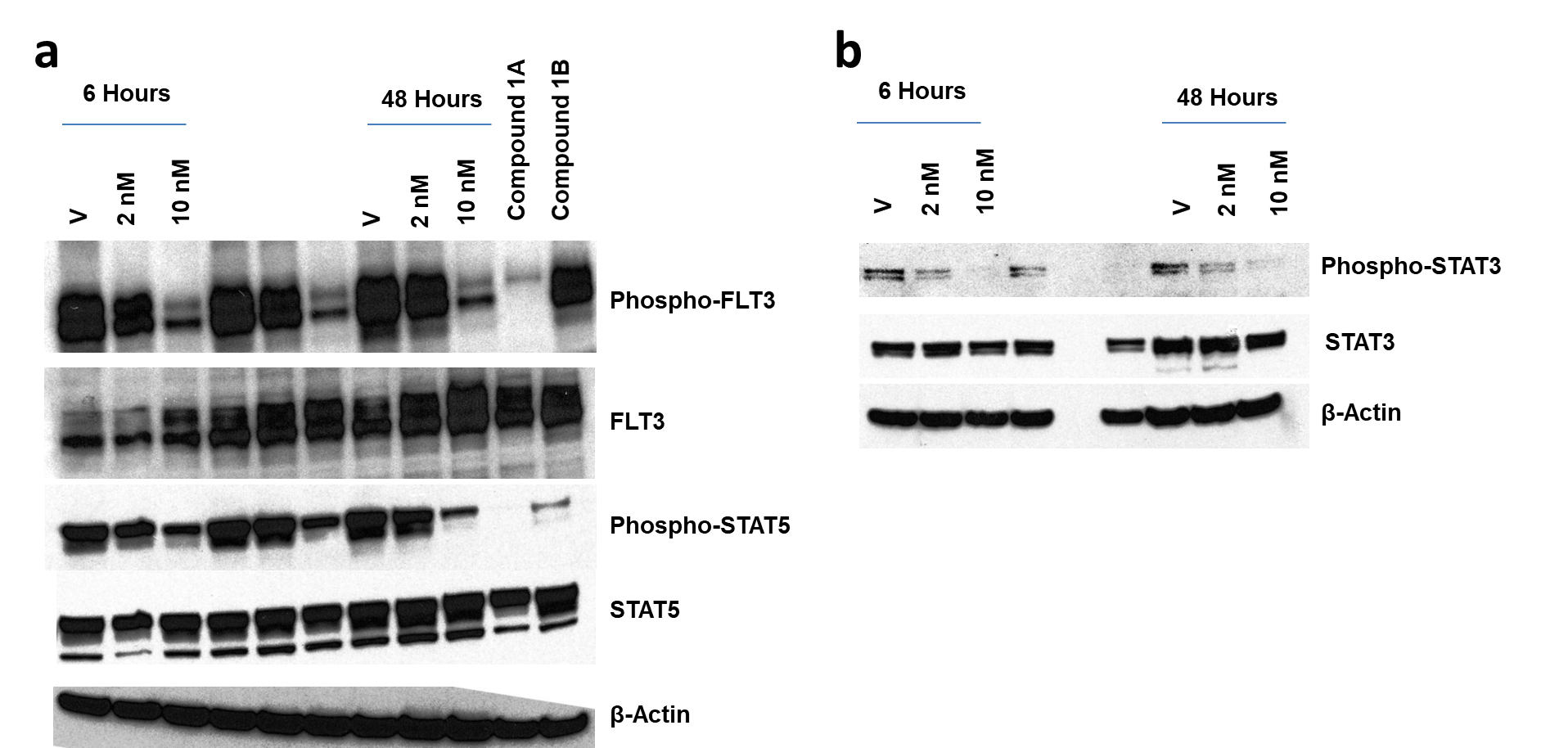


**Figure S6**. Full immunoblots for data presented in **Figure 6**. After transfer, immunoblots were cut into strips, probed with antibodies according to molecular weight ranges. Full immunoblots have been shown for full disclosure but compound 1A and 1B for example are for another study.


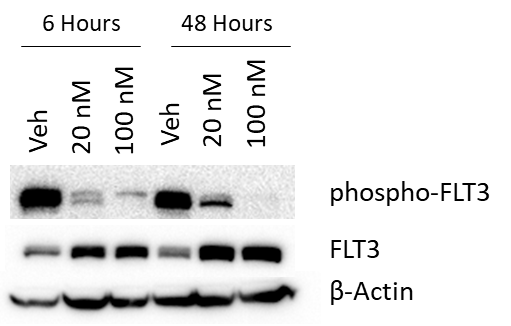


**Figure S7**. Effect of HSN431 on FLT3 phosphorylation in MOLM14 (ITD, D835Y)


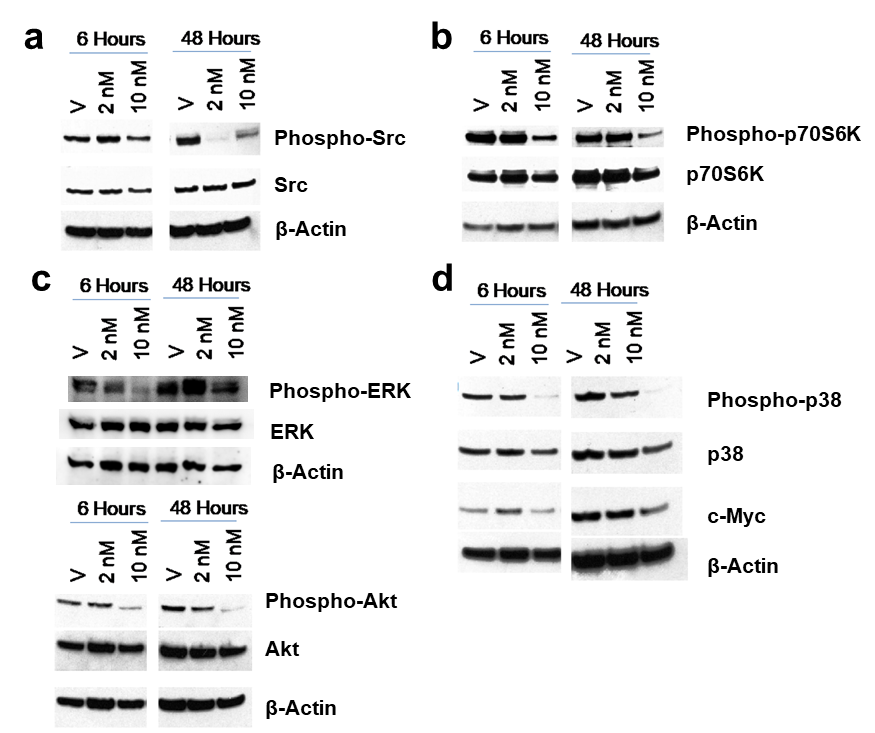


**Figure S8**. Western blot analysis showing the effect of HSN431 on the phosphorylation of various proteins in MV4-11. Cells were treated with 2 nM or 10 nM HSN431 for 6 h and 48 h, and protein extracts were probed with primary antibodies of (a) phospho-Src and Src, (b) phospho-p70S6K and p70S6K, (c) phospho-ERK, ERK, phospho-Akt and Akt, and (d) phospho-p38, p38 and c-Myc. Primary antibodies of indicated kinases or proteins were detected using anti-rabbit or anti-mouse secondary antibodies. Scanned images were analyzed using image J software.

**
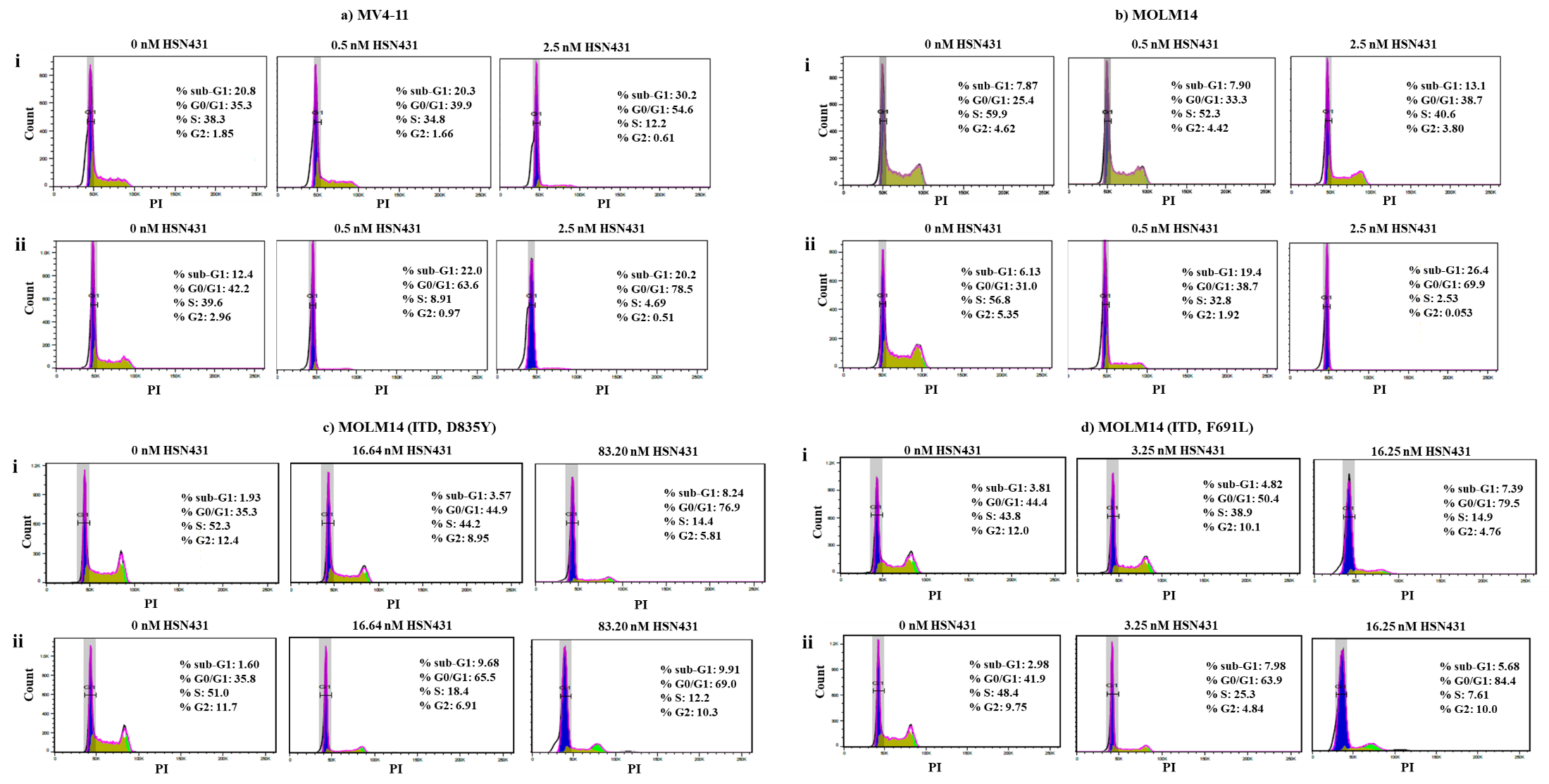
**

**Figure S9**. Effect of HSN431 on the cell cycle distribution in the AML cell lines (a) MV4-11, (b) MOLM14, (c) MOLM14 (D835Y) and (d) MOLM14 (ITD, F691L) cells after incubating with the indicated concentrations of HSN431 for (i) 24 hours and (ii) 48 hours. Experiment was performed in triplicates and representative cytograms have been shown.


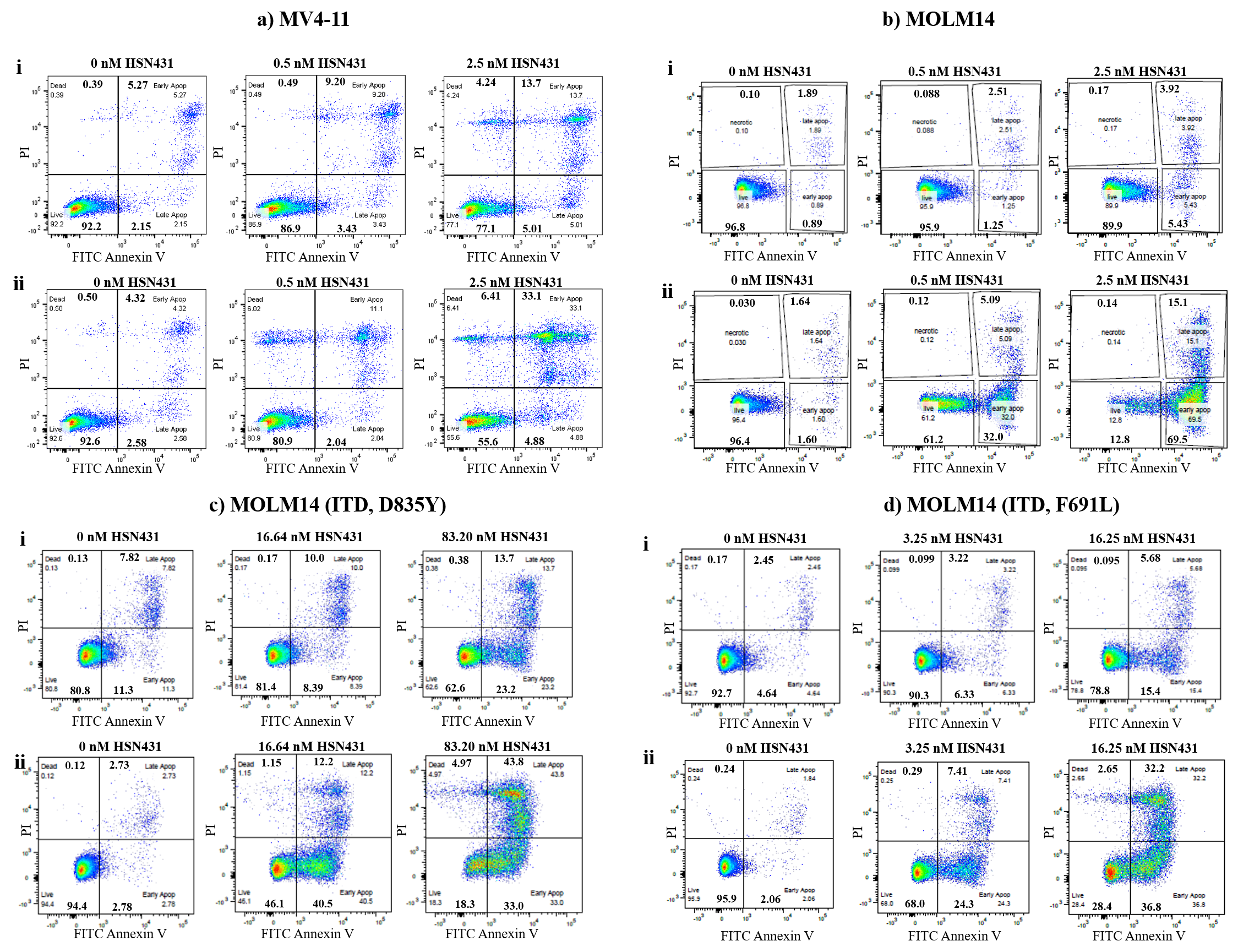


**Figure S10**. Representative data for apoptosis in AML cell lines. Apoptosis in (a) MV4-11, (b) MOLM14, (c) MOLM14 (ITD, D835Y) and (d) MOLM14 (ITD, F691L) cells after culturing for (i) 24 hours and (ii) 48 hours. The concentrations of HSN431 used are indicated on top of each figure.

**Supplementary methods**

**Chemistry**

Unless stated otherwise, all the chemicals required for synthesis were purchased from commercially available suppliers and used without further purification. All Sonogashira reactions were performed under an inert atmosphere of argon with magnetic stirring. Analytical thin layer chromatography (TLC) was performed on MERCK precoated silica gel 60 F254TLC plates. Eluting solvents are reported as volume percents. Compounds were visualized using UV light, Combi Flash was used for purification of compounds. All NMR spectra were recorded on Bruker 500 MHs spectrometers using CD3OD or DMSO-d6 as solvent. The NMR spectra were referenced using residual solvent peaks as the standard. Chemical shifts are denoted in parts per million (δ) and coupling constants (J) are reported in Hertz (Hz). The spin multiplicities are reported as singlet (s), broad singlet (bs), doublet (d), triplet (t), quartet (q), quintet (quint), apparent quintet (app. quint.) and multiplet (m).

- - - 1. **Synthesis and Analytical Data of Compounds**

**Compound 1: 3-((2-aminopyridin-3-yl)ethynyl)-4-methyl-N-(4-((4-methylpiperazin-1-yl)methyl)-3-(trifluoromethyl)phenyl)benzamide:** A solution of Iodo compound **1a** (80 mg, 0.36 mmol, 1 equiv), Pd(PPh_3_)_4_ (10 mol%), CuI (5 mol%) and Triphenylphosphine (10 mg) in Triethylamine (1 mL, 7.2 mmol, 20 equiv) was de-oxygenated using steam of Argon gas. A de-oxygenated solution of alkyne **1b** (150 mg, 0.36 mmol, 1 equiv) in DMF (4 mL) was added slowly over a period of 10 min to the solution and the reaction temperature was increased to 50 ºC and allowed to stir 8 h. The reaction was quenched by addition of NH_4_Cl (5 mL) at room temperature and diluted with ethyl acetate (300 mL). The organic layer was washed with water (5 × 50 mL) and washed with brine (1 × 50 mL). Combined organic layers were dried over anhydrous sodium sulphate, filtered and concentrated in vacuo. The pure product **1** was obtained by flash column chromatography. **Yield** = 77%.; **TLC R*_f_*** = 0.5 (10 % MeOH/CH_2_Cl_2_)

**^1^H NMR** (500 MHz, DMSO-*d*_6_) δ 10.50 (s, 1H), 8.23 (d, *J* = 1.9 Hz, 1H), 8.19 (d, *J* = 2.2 Hz, 1H), 8.04 (dd, *J* = 8.5, 2.2 Hz, 1H), 7.98 (dd, *J* = 4.9, 1.9 Hz, 1H), 7.86 (dd, *J* = 7.9, 2.0 Hz, 1H), 7.69 (d, *J* = 8.5 Hz, 1H), 7.63 (dd, *J* = 7.5, 1.9 Hz, 1H), 7.47 (d, *J* = 8.1 Hz, 1H), 6.59 (dd, *J* = 7.5, 4.9 Hz, 1H), 6.29 (s, 2H), 3.55 (s, 2H), 2.53 (s, 3H), 2.37 (s, 8H), 2.14 (s, 3H); **^13^C NMR** (126 MHz, DMSO) δ 165.38, 159.87, 149.05, 143.81, 140.44, 138.65, 132.63, 132.50, 131.69, 131.35, 130.18, 128.28, 125.88, 123.92, 123.05, 117.67, 112.62, 101.41, 93.06, 90.48, 57.90, 55.17, 53.14, 46.16, 20.99; **HRMS (ESI^+^):** calcd. for C_28_H_29_F_3_N_5_O (MH^+^) 508.2319, found 508.2315.

**Compound 2: 3-((6-aminopyridin-3-yl)ethynyl)-4-methyl-N-(4-((4-methylpiperazin-1-yl)methyl)-3-(trifluoromethyl)phenyl)benzamide:** A solution of Bromo compound **2a** (100 mg, 0.58 mmol, 1.5 equiv), Pd(PPh_3_)_4_ (10 mol%), CuI (5 mol%) and Triphenylphosphine (10 mg) in Triethylamine (1 mL, 7.6 mmol, 20 equiv) was de-oxygenated using steam of Argon gas. A de-oxygenated solution of alkyne **1b** (160 mg, 0.38 mmol, 1 equiv) in DMF (4 mL) was added slowly over a period of 10 min to the solution and the reaction temperature was increased to 50 ºC and allowed to stir 12 h. The reaction was quenched by addition of NH_4_Cl (5 mL) at room temperature and diluted with ethyl acetate (300 mL). The organic layer was washed with water (5 × 50 mL) and washed with brine (1 × 50 mL). Combined organic layers were dried over anhydrous sodium sulphate, filtered and concentrated in vacuo. The pure product **2** was obtained by flash column chromatography. **Yield** = 57%.; **TLC R*_f_*** = 0.5 (10 % MeOH/CH_2_Cl_2_)

**^1^H NMR** (500 MHz, DMSO-*d*_6_) δ 10.48 (s, 1H), 8.19 (d, *J* = 2.2 Hz, 1H), 8.15 (d, *J* = 2.3 Hz, 1H), 8.07 (d, *J* = 2.0 Hz, 1H), 8.04 (dd, *J* = 8.5, 2.2 Hz, 1H), 7.84 (dd, *J* = 8.0, 2.0 Hz, 1H), 7.68 (d, *J* = 8.5 Hz, 1H), 7.53 (dd, *J* = 8.5, 2.4 Hz, 1H), 7.46 (d, *J* = 8.1 Hz, 1H), 6.47 (s, 1H), 6.45 (s, 2H), 3.54 (s, 2H), 2.49 (s, 3H), 2.36 (s, 8H), 2.15 (s, 3H); **^13^C NMR** (126 MHz, DMSO) δ 165.24, 159.84, 151.70, 143.52, 139.83, 138.65, 132.51, 131.67, 130.44, 130.27, 127.91, 123.94, 123.41, 117.70, 108.17, 106.39, 93.53, 87.65, 57.90, 55.16, 53.11, 46.14, 20.89; **HRMS (ESI^+^):** calcd. for C_28_H_29_F_3_N_5_O (MH^+^) 508.2319, found 508.2308.

**Compound 3: 3-((3-amino-6-bromopyrazin-2-yl)ethynyl)-4-methyl-N-(4-((4-methylpiperazin-1-yl)methyl)-3-(trifluoromethyl)phenyl)benzamide:** A solution of Iodo compound **4a** (99 mg, 0.33 mmol, 1 equiv), Pd(PPh_3_)_2_ Cl_2_ (5 mol%), CuI (5 mol%) and Triphenylphosphine (10 mg) in Triethylamine (1 mL, 7.2 mmol, 22 equiv) was de-oxygenated using steam of Argon gas. A de-oxygenated solution of alkyne **1b** (0.4 mmol, 1.2 equiv) in DMF (3 mL) was added slowly over a period of 10 min to the solution and the reaction temperature was increased to 50 ºC and allowed to stir 12 h. The reaction was quenched by addition of NH_4_Cl (5 mL) at room temperature. The crude compound was extracted using EtOAc (3 × 40 mL). Combined organic layers were dried over anhydrous sodium sulphate, filtered and concentrated in vacuo. The pure product **5** was obtained by flash column chromatography. **Yield** = 38%, **TLC R*_f_*** = 0.3 (10 % MeOH/CH_2_Cl_2_)

**^1^H NMR** (500 MHz, MeOD) δ 8.23 (d, *J* = 1.5 Hz, 1H), 8.14 (d, *J* = 1.7 Hz, 1H), 8.07 (s, 1H), 7.97 – 7.90 (m, 2H), 7.76 (d, *J* = 8.5 Hz, 1H), 7.47 (d, *J* = 8.1 Hz, 1H), 3.68 (s, 2H), 2.75 – 2.54 (m, 11H), 2.42 (s, 3H); **^13^C NMR** (126 MHz, MeOD) δ 166.15, 155.32, 144.72, 143.96, 137.87, 132.42, 132.30, 131.29, 131.18, 129.77, 128.45, 123.74, 123.59, 123.28, 121.65, 117.77, 117.72, 94.05, 87.62, 57.37, 54.42, 51.82, 48.45, 44.15, 19.61; **HR-MS(ESI)** m/z calcd for C_27_H_26_BrF_3_N_6_O ([M+H]+) 588.4332, found 588.433

**Compound 4^1^**

**Compound 5: 5-((3-aminoisoquinolin-4-yl)ethynyl)-6-methyl-N-(4-((4-methylpiperazin-1-yl)methyl)-3-(trifluoromethyl)phenyl)nicotinamide:** A solution of Iodo compound **5a** (70 mg, 0.26 mmol, 1 equiv), Pd(PPh_3_)_2_Cl_2_ (10 mol%), CuI (5 mol%) and Triphenylphosphine (10 mg) in Triethylamine (0.9 mL, 6.5 mmol, 20 equiv) was de-oxygenated using steam of Argon gas. A de-oxygenated solution of alkyne **2b** (129 mg, 0.31 mmol, 1.2 equiv) in DMF (4 mL) was added slowly over a period of 10 min to the solution and the reaction temperature was increased to 50 ºC and allowed to stir 6 h. The reaction was quenched by addition of NH_4_Cl (5 mL) at room temperature and diluted with ethyl acetate (300 mL). The organic layer was washed with water (5 × 50 mL) and washed with brine (1 × 50 mL). Combined organic layers were dried over anhydrous sodium sulphate, filtered and concentrated in vacuo. The pure product **7** was obtained by flash column chromatography. **Yield** = 45%; **TLC R*_f_*** = 0.3 (10 % MeOH/CH_2_Cl_2_)

**^1^H NMR** (500 MHz, DMSO-*d*_6_) δ 10.70 (s, 1H), 8.94 (d, *J* = 2.2 Hz, 1H), 8.91 (s, 1H), 8.69 (d, *J* = 2.3 Hz, 1H), 8.19 (d, *J* = 2.2 Hz, 1H), 8.04 (dd, *J* = 8.5, 2.2 Hz, 1H), 7.96 – 7.89 (m, 2H), 7.74 – 7.66 (m, 2H), 7.29 (ddd, *J* = 8.0, 6.8, 1.1 Hz, 1H), 6.69 (s, 2H), 3.56 (s, 2H), 2.85 (s, 3H), 2.37 (s, 8H), 2.15 (s, 3H); **^13^C NMR** (126 MHz, DMSO) δ 164.32, 162.08, 158.37, 153.61, 147.14, 138.49, 138.38, 137.91, 132.83, 132.33, 131.79, 129.23, 128.14, 128.03, 123.96, 123.17, 122.62, 122.37, 119.28, 117.70, 95.98, 91.74, 88.99, 57.89, 55.15, 53.11, 46.12, 24.48; **HRMS (ESI^+^):** calcd. for C_31_H_30_F_3_N_6_O (MH^+^) 559.2428, found 559.2433

**Compound 6: 3-((3-amino-6-fluoroisoquinolin-4-yl)ethynyl)-4-methyl-N-(4-((4-methylpiperazin-1-yl)methyl)-3-(trifluoromethyl)phenyl)benzamide:** A solution of Iodo compound **6a** (30 mg, 0.1 mmol, 1 equiv), Pd(PPh_3_)_2_ Cl_2_ (10 mol%), CuI (5 mol%) and Triphenylphosphine (4 mg) in Triethylamine (0.5 mL, 3 mmol, 30 equiv) was de-oxygenated using steam of Argon gas. A de-oxygenated solution of alkyne **1b** (65mg, 0.16 mmol, 1.5 equiv) in DMF (2 mL) was added slowly over a period of 10 min to the solution and the reaction temperature was increased to 50 ºC and allowed to stir 12 h. The reaction was quenched by addition of NH_4_Cl (2 mL) at room temperature and diluted with ethyl acetate (100 mL). The organic layer was washed with water (5 × 30 mL) and washed with brine (20 mL). Combined organic layers were dried over anhydrous sodium sulphate , filtered and concentrated in vacuo. The pure product **8** was obtained by flash column chromatography. **Yield** = 60%; **TLC R*_f_*** = 0.2 (10 % MeOH/CH_2_Cl_2_)

**^1^H NMR** (500 MHz, Methanol-*d*_4_) δ 8.93 (s, 1H), 8.37 (d, *J* = 1.9 Hz, 1H), 8.25 (d, *J* = 2.2 Hz, 1H), 8.10 – 8.02 (m, 2H), 7.95 (dd, *J* = 8.0, 2.0 Hz, 1H), 7.81 (d, *J* = 8.4 Hz, 1H), 7.66 (dd, *J* = 10.6, 2.4 Hz, 1H), 7.57 (d, *J* = 8.0 Hz, 1H), 7.19 (td, *J* = 8.8, 2.5 Hz, 1H), 3.69 (s, 2H), 2.74 (s, 3H), 2.56 (s, 8H), 2.35 (s, 3H); **^13^C NMR** (126 MHz, MeOD) δ 165.94, 163.90, 157.90, 152.39, 143.40, 139.79, 138.30, 132.68, 132.55, 132.45, 132.37, 131.40, 130.88, 129.95, 127.77, 123.71, 123.45, 117.68, 113.24, 113.04, 109.71, 106.14, 105.96, 98.05, 88.03, 57.65, 54.78, 52.42, 44.91, 20.48; **HRMS (ESI^+^):** calcd. for C_32_H_30_F_4_N_5_O (MH^+^) 576.2386, found 576.2382

**Compound 7: 5-((3-amino-6-fluoroisoquinolin-4-yl)ethynyl)-6-methyl-N-(4-((4-methylpiperazin-1-yl)methyl)-3-(trifluoromethyl)phenyl)nicotinamide:** A solution of Iodo compound **6a** (80 mg, 0.28 mmol, 1 equiv), Pd(PPh_3_)_2_ Cl_2_ (10 mol%), CuI (5 mol%) and Triphenylphosphine (10 mg) in Triethylamine (1.2 mL, 8.4 mmol, 30 equiv) was de-oxygenated using steam of Argon gas. A de-oxygenated solution of alkyne **2b** (139mg, 0.33 mmol, 1.2 equiv) in DMF (3 mL) was added slowly over a period of 10 min to the solution and the reaction temperature was increased to 50 ºC and allowed to stir 4 h. The reaction was quenched by addition of NH_4_Cl (5 mL) at room temperature and diluted with ethyl acetate (300 mL). The organic layer was washed with water (5 × 50 mL) and washed with brine (50 mL). Combined organic layers were dried over anhydrous sodium sulphate , filtered and concentrated in vacuo. The pure product **9** was obtained by flash column chromatography. **Yield** = 52%.; **TLC R*_f_*** = 0.2 (10 % MeOH/CH_2_Cl_2_)

**^1^H NMR** (500 MHz, DMSO-*d*_6_) δ 10.70 (s, 1H), 8.94 (d, *J* = 2.2 Hz, 1H), 8.91 (s, 1H), 8.70 (d, *J* = 2.3 Hz, 1H), 8.19 (d, *J* = 2.2 Hz, 1H), 8.03 (m, 2H), 7.72 (d, *J* = 8.5 Hz, 1H), 7.52 (dd, *J* = 10.8, 2.5 Hz, 1H), 7.17 (td, *J* = 8.8, 2.5 Hz, 1H), 6.86 (s, 2H), 3.56 (s, 2H), 2.84 (s, 3H), 2.38 (s, 8H), 2.16 (s, 3H); **^13^C NMR** (126 MHz, DMSO) δ 164.31, 162.06, 158.77, 153.56, 147.19, 139.69, 138.59, 138.38, 132.83, 131.78, 128.14, 123.96, 119.73, 119.17, 117.69, 113.21, 113.00, 106.20, 106.02, 96.22, 91.09, 88.79, 57.88, 55.15, 53.10, 46.11, 24.42; **HRMS (ESI^+^):** calcd. for C_31_H_29_F_4_N_6_O (MH^+^) 577.2339, found 577.2333

**Compound 8^1^**

**Compound 9: 5-((3-amino-6-chloroisoquinolin-4-yl)ethynyl)-6-methyl-N-(4-((4-methylpiperazin-1-yl)methyl)-3-(trifluoromethyl)phenyl)nicotinamide:** A solution of Bromo compound **7a** (97 mg, 0.21 mmol, 1 equiv), Pd(PPh_3_)_2_ Cl_2_ (5 mol%), CuI (5 mol%) and Triphenylphosphine (5 mg) in Triethylamine (1 mL, 7.5 mmol, 30 equiv) was de-oxygenated using steam of Argon gas. A de-oxygenated solution of alkyne **3b** (50 mg, 0.25 mmol, 1.2 equiv)in DMF (3 mL) was added slowly over a period of 10 min to the solution and the reaction temperature was increased to 50 ºC and allowed to stir 12 h. The reaction was quenched by addition of NH_4_Cl (5 mL) at room temperature. The crude compound was extracted using EtOAc (3 × 30 mL). Combined organic layers were dried over anhydrous sodium sulphate , filtered and concentrated in vacuo. The pure product **11** was obtained by flash column chromatography. **Yield** = 52%; **TLC R*_f_*** = 0.2 (10 % MeOH/CH_2_Cl_2_)

**^1^H NMR** (500 MHz, DMSO-*d*_6_) δ 10.71 (s, 1H), 8.95 (d, *J* = 2.3 Hz, 1H), 8.94 (s, 1H), 8.71 (d, *J* = 2.3 Hz, 1H), 8.19 (d, *J* = 2.2 Hz, 1H), 8.04 (dd, *J* = 8.5, 2.2 Hz, 1H), 7.96 (d, *J* = 8.6 Hz, 1H), 7.87 (d, *J* = 2.0 Hz, 1H), 7.72 (d, *J* = 8.5 Hz, 1H), 7.29 (dd, *J* = 8.6, 2.0 Hz, 1H), 6.92 (s, 2H), 3.56 (s, 2H), 2.84 (s, 3H), 2.38 (s, 8H), 2.17 (s, 3H); **^13^C NMR** (126 MHz, DMSO) δ 164.32, 162.10, 158.88, 153.71, 147.25, 138.80, 138.65, 138.39, 137.55, 132.83, 131.81, 131.70, 128.17, 127.81, 125.85, 123.97, 123.55, 121.28, 120.62, 119.14, 117.69, 96.27, 90.91, 88.11, 57.88, 55.13, 53.06, 46.07, 24.37; **HRMS (ESI^+^):** calcd. for C_31_H_29_ClF_3_N_6_O (MH^+^) 593.2043, found 593.2036

**Compound 10:** **4-((3-amino-6-chloroisoquinolin-4-yl)ethynyl)-5-methyl-N-(4-((4-methylpiperazin-1-yl)methyl)-3-(trifluoromethyl)phenyl)picolinamide:** A solution of Bromo compound **8a** (58 mg, 0.12 mmol, 1 equiv), Pd(PPh_3_)_2_ Cl_2_ (10 mol%), CuI (5 mol%) and Triphenylphosphine (5 mg) in Triethylamine (0.6 mL, 4.5 mmol, 30 equiv) was de-oxygenated using steam of Argon gas. A de-oxygenated solution of alkyne **3b** (30mg, 0.15 mmol, 1.2 equiv) in DMF (2 mL) was added slowly over a period of 10 min to the solution and the reaction temperature was increased to 45 ºC and allowed to stir 12 h. The reaction was quenched by addition of NH_4_Cl (2 mL) at room temperature and diluted with ethyl acetate (100 mL). The organic layer was washed with water (5 × 30 mL) and washed with brine (20 mL). Combined organic layers were dried over anhydrous sodium sulphate , filtered and concentrated in vacuo. The pure product **12** was obtained by flash column chromatography. **Yield** = 51%; **TLC R*_f_*** = 0.2 (10 % MeOH/CH_2_Cl_2_)

**^1^H NMR** (500 MHz, DMSO-*d*_6_) δ 10.94 (s, 1H), 8.96 (s, 1H), 8.69 (s, 1H), 8.57 (s, 1H), 8.38 (d, *J* = 2.2 Hz, 1H), 8.16 (d, *J* = 7.9 Hz, 1H), 7.97 (d, *J* = 8.6 Hz, 1H), 7.87 (s, 1H), 7.69 (d, *J* = 8.5 Hz, 1H), 7.29 (dd, *J* = 8.6, 2.0 Hz, 1H), 7.11 (s, 2H), 3.55 (s, 2H), 2.65 (s, 3H), 2.38 (s, 8H), 2.16 (s, 3H); **^13^C NMR** (126 MHz, DMSO) δ 163.27, 159.25, 154.54, 149.38, 148.11, 139.01, 138.02, 137.74, 137.12, 132.94, 132.73, 131.78, 131.60, 124.35, 124.17, 123.57, 121.26, 120.59, 117.99, 96.18, 94.13, 87.32, 57.90, 55.17, 53.11, 46.12, 18.19; **HRMS (ESI^+^):** calcd. for C_31_H_29_ClF_3_N_6_O (MH^+^) 593.2043, found 593.2039

**Compound 11: 6-((3-amino-6-chloroisoquinolin-4-yl)ethynyl)-5-methyl-N-(4-((4-methylpiperazin-1-yl)methyl)-3-(trifluoromethyl)phenyl)picolinamide:** A solution of Bromo compound **9a** (58 mg, 0.12 mmol, 1 equiv), Pd(PPh_3_)_2_ Cl_2_ (10 mol%), CuI (5 mol%) and Triphenylphosphine (5 mg) in Triethylamine (0.6 mL, 4.5 mmol, 30 equiv) was de-oxygenated using steam of Argon gas. A de-oxygenated solution of alkyne **3b** (30mg, 0.15 mmol, 1.2 equiv) in DMF (2 mL) was added slowly over a period of 10 min to the solution and the reaction temperature was increased to 45 ºC and allowed to stir 12 h. The reaction was quenched by addition of NH_4_Cl (2 mL) at room temperature and diluted with ethyl acetate (100 mL). The organic layer was washed with water (5 × 30 mL) and washed with brine (20 mL). Combined organic layers were dried over anhydrous sodium sulphate , filtered and concentrated in vacuo. The pure product **13** was obtained by flash column chromatography. **Yield** = 44%; **TLC R*_f_*** = 0.2 (10 % MeOH/CH_2_Cl_2_)

**^1^H NMR** (500 MHz, DMSO-*d*_6_) δ 10.85 (s, 1H), 8.98 (s, 1H), 8.35 (d, *J* = 2.2 Hz, 1H), 8.17 (d, *J* = 8.5 Hz, 1H), 8.06 – 8.00 (m, 2H), 7.99 (d, *J* = 8.6 Hz, 1H), 7.94 (s, 1H), 7.72 (d, *J* = 8.6 Hz, 1H), 7.31 (dd, *J* = 8.6, 2.0 Hz, 1H), 6.88 (s, 2H), 3.57 (s, 2H), 2.66 (s, 3H), 2.38 (s, 8H), 2.16 (s, 3H); **^13^C NMR** (126 MHz, DMSO) δ 163.02, 159.17, 154.17, 148.08, 141.92, 139.53, 139.39, 138.90, 137.89, 137.71, 132.82, 131.75, 131.67, 124.16, 123.76, 121.94, 121.42, 120.71, 117.96, 97.81, 88.52, 87.59, 57.90, 55.13, 53.07, 46.07, 19.94; **HRMS (ESI^+^):** calcd. for C_31_H_29_ClF_3_N_6_O (MH^+^) 593.2043, found 593.2039

**Compound 12: 3-((3-amino-6-methylisoquinolin-4-yl)ethynyl)-4-methyl-N-(4-((4-methylpiperazin-1-yl)methyl)-3-(trifluoromethyl)phenyl)benzamide:** A solution of Iodo compound **10a** (60 mg, 0.2 mmol, 1 equiv), Pd(PPh_3_)_2_ Cl_2_ (10 mol%), CuI (5 mol%) and Triphenylphosphine (5 mg) in Triethylamine (0.83 mL, 6 mmol, 30 equiv) was de-oxygenated using steam of Argon gas. A de-oxygenated solution of alkyne **1b** (105mg, 0.25 mmol, 1.2 equiv) in DMF (3 mL) was added slowly over a period of 10 min to the solution and the reaction temperature was increased to 55 ºC and allowed to stir 12 h. The reaction was quenched by addition of NH_4_Cl (5 mL) at room temperature. The crude compound was extracted using EtOAc (3 × 30 mL). Combined organic layers were dried over anhydrous sodium sulphate, filtered and concentrated in vacuo. The pure product **14** was obtained by flash column chromatography. **Yield** = 54%.; **TLC R*_f_*** = 0.2 (10 % MeOH/CH_2_Cl_2_)

**^1^H NMR** (500 MHz, DMSO-*d*_6_) δ 10.54 (s, 1H), 8.81 (d, *J* = 0.7 Hz, 1H), 8.35 (d, *J* = 2.0 Hz, 1H), 8.21 (d, *J* = 2.2 Hz, 1H), 8.05 (dd, *J* = 8.5, 2.2 Hz, 1H), 7.87 (dd, *J* = 8.0, 2.0 Hz, 1H), 7.80 (d, *J* = 8.3 Hz, 1H), 7.74 – 7.72 (m, 1H), 7.70 (d, *J* = 8.6 Hz, 1H), 7.51 (d, *J* = 8.1 Hz, 1H), 7.13 (dd, *J* = 8.3, 1.5 Hz, 1H), 6.48 (s, 2H), 3.56 (s, 2H), 2.65 (s, 3H), 2.47 (s, 3H), 2.37 (bs, 8H), 2.15 (s, 3H); **^13^C NMR** (126 MHz, DMSO) δ 165.58, 158.09, 152.60, 143.24, 142.22, 138.70, 138.16, 132.79, 132.50, 131.72, 131.33, 130.21, 129.01, 127.88, 125.91, 125.34, 123.94, 123.74, 121.73, 120.96, 117.69, 97.80, 89.57, 89.32, 57.92, 55.20, 53.16, 46.19, 22.50, 21.33; **HRMS (ESI^+^):** calcd. for C_33_H_32_F_3_N_5_O (MH^+^) 572.2637, found 572.2645

**Compound 13: 5-((3-amino-6-methylisoquinolin-4-yl)ethynyl)-6-methyl-N-(4-((4-methylpiperazin-1-yl)methyl)-3-(trifluoromethyl)phenyl)nicotinamide:** A solution of Iodo compound **10a** (51 mg, 0.18 mmol, 1 equiv), Pd(PPh_3_)_2_ Cl_2_ (10 mol%), CuI (5 mol%) and Triphenylphosphine (10 mg) in Triethylamine (0.75 mL, 5.4 mmol, 30 equiv) was de-oxygenated using steam of Argon gas. A de-oxygenated solution of alkyne **2b** (90mg, 0.22 mmol, 1.2 equiv) in DMF (3 mL) was added slowly over a period of 10 min to the solution and the reaction temperature was increased to 50 ºC and allowed to stir 12 h. The reaction was quenched by addition of NH_4_Cl (5 mL) at room temperature and diluted with ethyl acetate (250 mL). The organic layer was washed with water (5 × 50 mL) and washed with brine (50 mL). Combined organic layers were dried over anhydrous sodium sulphate, filtered and concentrated in vacuo. The pure product **14** was obtained by flash column chromatography. **Yield** = 58%.; **TLC R*_f_*** = 0.2 (10 % MeOH/CH_2_Cl_2_)

**^1^H NMR** (500 MHz, DMSO-*d*_6_) δ 10.71 (s, 1H), 8.93 (d, *J* = 2.3 Hz, 1H), 8.82 (s, 1H), 8.66 (d, *J* = 2.2 Hz, 1H), 8.18 (d, *J* = 2.3 Hz, 1H), 8.02 (dd, *J* = 8.5, 2.2 Hz, 1H), 7.81 (d, *J* = 8.3 Hz, 1H), 7.71 (d, *J* = 9.0 Hz, 2H), 7.13 (dd, *J* = 8.3, 1.5 Hz, 1H), 6.60 (s, 2H), 3.56 (s, 2H), 2.85 (s, 3H), 2.47 (s, 3H), 2.37 (bs, 8H), 2.15 (s, 3H); **^13^C NMR** (126 MHz, DMSO) δ 164.37, 162.11, 158.39, 153.12, 147.06, 142.48, 138.44, 138.35, 138.20, 132.86, 131.84, 129.10, 128.15, 125.40, 124.00, 121.64, 120.88, 119.37, 117.73, 95.90, 91.91, 88.65, 57.87, 55.11, 53.04, 46.06, 24.38, 22.50.

**Compound 14:** **5-((1-amino-6-methoxyisoquinolin-4-yl)ethynyl)-6-methyl-N-(4-((4-methylpiperazin-1-yl)methyl)-3-(trifluoromethyl)phenyl)nicotinamide:** A solution of Bromo compound **11a** (139 mg, 0.55 mmol, 1.5 equiv), Pd(PPh_3_)_4_ (10 mol%), CuI (5 mol%) and Triphenylphosphine (10 mg) in Triethylamine (1 mL, 7.2 mmol, 20 equiv) was de-oxygenated using steam of Argon gas. A de-oxygenated solution of alkyne **2b** (152 mg, 0.36 mmol, 1 equiv) in DMF (4 mL) was added slowly over a period of 10 min to the solution and the reaction temperature was increased to 50 ºC and allowed to stir 12 h. The reaction was quenched by addition of NH_4_Cl (5 mL) at room temperature and diluted with ethyl acetate (300 mL). The organic layer was washed with water (5 × 50 mL) and washed with brine (1 × 50 mL). Combined organic layers were dried over anhydrous sodium sulphate, filtered and concentrated in vacuo. The pure product **16** was obtained by flash column chromatography. **Yield** = 52%.; **TLC R*_f_*** = 0.3 (10 % MeOH/CH_2_Cl_2_)

**^1^H NMR** (500 MHz, DMSO-*d*_6_) δ 10.66 (s, 1H), 8.92 (d, *J* = 2.3 Hz, 1H), 8.44 (d, *J* = 2.3 Hz, 1H), 8.22 (d, *J* = 9.1 Hz, 1H), 8.19 (d, *J* = 2.2 Hz, 1H), 8.17 (s, 1H), 8.03 (dd, *J* = 8.5, 2.2 Hz, 1H), 7.71 (d, *J* = 8.5 Hz, 1H), 7.40 (d, *J* = 2.6 Hz, 1H), 7.31 (s, 2H), 7.18 (dd, *J* = 9.1, 2.6 Hz, 1H), 3.94 (s, 3H), 3.56 (s, 2H), 2.84 (s, 3H), 2.37 (s, 8H), 2.14 (s, 3H); **^13^C NMR** (126 MHz, DMSO) δ 164.08, 162.00, 161.69, 158.20, 148.87, 147.12, 138.69, 138.37, 137.41, 132.86, 131.78, 127.96, 127.19, 123.97, 119.28, 117.79, 111.31, 104.07, 102.28, 94.68, 90.26, 57.91, 55.84, 55.20, 53.18, 46.20, 24.32; **HRMS (ESI^+^):** calcd. for C_32_H_32_F_3_N_6_O_2_ (MH^+^) 589.2533, found 589.2538

**Compound 15^1^**

**Compound 16: 5-((1-aminoisoquinolin-4-yl)ethynyl)-6-methyl-N-(4-((4-methylpiperazin-1-yl)methyl)-3-(trifluoromethyl)phenyl)nicotinamide:** A solution of Bromo compound **12a** (77 mg, 0.35 mmol, 1.2 equiv), Pd(PPh_3_)_2_ Cl_2_ (10 mol%), CuI (5 mol%) and Triphenylphosphine (10 mg) in Triethylamine (1 mL, 7 mmol, 20 equiv) was de-oxygenated using steam of Argon gas. A de-oxygenated solution of alkyne **2b** (120mg, 0.29 mmol, 1 equiv) in DMF (3 mL) was added slowly over a period of 10 min to the solution and the reaction temperature was increased to 50 ºC and allowed to stir 12 h. The reaction was quenched by addition of NH_4_Cl (5 mL) at room temperature and diluted with ethyl acetate (300 mL). The organic layer was washed with water (5 × 50 mL) and washed with brine (50 mL). Combined organic layers were dried over anhydrous sodium sulphate, filtered and concentrated in vacuo. The pure product **18** was obtained by flash column chromatography. **Yield** = 31%.; **TLC R*_f_*** = 0.2 (10 % MeOH/CH_2_Cl_2_)

**^1^H NMR** (500 MHz, DMSO-*d*_6_) δ 10.66 (s, 1H), 8.93 (d, *J* = 2.3 Hz, 1H), 8.48 (d, *J* = 2.3 Hz, 1H), 8.29 (d, *J* = 8.1 Hz, 1H), 8.22 (s, 1H), 8.20 (d, *J* = 2.2 Hz, 1H), 8.11 (d, *J* = 8.1 Hz, 1H), 8.04 (dd, *J* = 8.4, 2.2 Hz, 1H), 7.81 (ddd, *J* = 8.1, 6.9, 1.2 Hz, 1H), 7.72 (d, *J* = 8.5 Hz, 1H), 7.58 (ddd, *J* = 8.3, 6.9, 1.3 Hz, 1H), 7.45 (s, 2H), 3.56 (s, 2H), 2.81 (s, 3H), 2.37 (s, 8H), 2.14 (s, 3H); **^13^C NMR** (126 MHz, DMSO) δ 164.08, 162.21, 158.42, 148.15, 147.27, 138.36, 137.64, 136.33, 132.87, 131.78, 131.71, 127.95, 126.86, 125.03, 124.74, 124.00, 119.15, 117.74, 116.71, 102.62, 94.26, 89.98, 57.91, 55.20, 53.18, 46.21, 24.30; **HRMS (ESI^+^):** calcd. for C_31_H_30_F_3_N_6_O (MH^+^) 559.2433, found 559.2430

**Compound 17: 3-((1-amino-6-fluoroisoquinolin-4-yl)ethynyl)-4-methyl-N-(4-((4-methylpiperazin-1-yl)methyl)-3-(trifluoromethyl)phenyl)benzamide:** A solution of Bromo compound **13a** (174 mg, 0.72 mmol, 1.5 equiv), Pd(PPh_3_)_4_ (10 mol%), CuI (5 mol%) and Triphenylphosphine (10 mg) in Triethylamine (1.3 mL, 9.6 mmol, 20 equiv) was de-oxygenated using steam of Argon gas. A de-oxygenated solution of alkyne **1b** (200 mg, 0.48 mmol, 1 equiv) in DMF (4 mL) was added slowly over a period of 10 min to the solution and the reaction temperature was increased to 50 ºC and allowed to stir 12 h. The reaction was quenched by addition of NH_4_Cl (5 mL) at room temperature and diluted with ethyl acetate (300 mL). The organic layer was washed with water (5 × 50 mL) and washed with brine (1 × 50 mL). Combined organic layers were dried over anhydrous sodium sulphate, filtered and concentrated in vacuo. The pure product **19** was obtained by flash column chromatography. **Yield** = 47%; **TLC R*_f_*** = 0.3 (10 % MeOH/CH_2_Cl_2_)

**^1^H NMR** (500 MHz, DMSO-*d*_6_) δ 10.52 (s, 1H), 8.40 (dd, *J* = 9.2, 5.5 Hz, 1H), 8.21 (s, 2H), 8.19 (d, *J* = 1.9 Hz, 1H), 8.05 (dd, *J* = 8.5, 2.2 Hz, 1H), 7.87 (dd, *J* = 7.9, 2.0 Hz, 1H), 7.75 – 7.65 (m, 2H), 7.49 (m, 4H), 3.54 (s, 2H), 2.59 (s, 3H), 2.37 (s, 8H), 2.14 (s, 3H); **^13^C NMR** (126 MHz, DMSO) δ 165.31, 162.91, 158.01, 148.90, 143.29, 138.67, 132.67, 132.47, 131.66, 130.68, 130.32, 127.96, 123.97, 123.50, 117.69, 116.17, 115.97, 113.81, 108.51, 102.92, 92.05, 91.25, 57.91, 55.16, 53.11, 46.12, 21.15; **HRMS (ESI^+^):** calcd. for C_32_H_30_F_4_N_5_O (MH^+^) 576.2381, found 576.2387

**Compound 18: 5-((3-amino-6-methoxyisoquinolin-4-yl)ethynyl)-6-methyl-N-(4-((4-methylpiperazin-1-yl)methyl)-3-(trifluoromethyl)phenyl)nicotinamide:** A solution of Iodo compound **14a** (100 mg, 0.33 mmol, 1 equiv), Pd(PPh_3_)_4_ (10 mol%), CuI (5 mol%) and Triphenylphosphine (10 mg) in Triethylamine (1.4 mL, 9.9 mmol, 20 equiv) was de-oxygenated using steam of Argon gas. A de-oxygenated solution of alkyne **2b** (166 mg, 0.4 mmol, 1.2 equiv) in DMF (4 mL) was added slowly over a period of 10 min to the solution and the reaction temperature was increased to 50 ºC and allowed to stir 12 h. The reaction was quenched by addition of NH_4_Cl (5 mL) at room temperature and diluted with ethyl acetate (300 mL). The organic layer was washed with water (5 × 50 mL) and washed with brine (1 × 50 mL). Combined organic layers were dried over anhydrous sodium sulphate, filtered and concentrated in vacuo. The pure product **20** was obtained by flash column chromatography. **Yield** = 69%.; **TLC R*_f_*** = 0.3 (10 % MeOH/CH_2_Cl_2_)

**^1^H NMR** (500 MHz, DMSO-*d*_6_) δ 10.71 (s, 1H), 8.92 (d, *J* = 2.3 Hz, 1H), 8.74 (s, 1H), 8.72 (d, *J* = 2.2 Hz, 1H), 8.19 (d, *J* = 2.2 Hz, 1H), 8.04 (dd, *J* = 8.5, 2.2 Hz, 1H), 7.81 (d, *J* = 8.9 Hz, 1H), 7.72 (d, *J* = 8.5 Hz, 1H), 7.21 (d, *J* = 2.3 Hz, 1H), 6.90 (dd, *J* = 8.9, 2.4 Hz, 1H), 6.69 (s, 2H), 3.92 (s, 3H), 3.56 (s, 2H), 2.87 (s, 3H), 2.39 (s, 8H), 2.17 (s, 3H); **^13^C NMR** (126 MHz, DMSO) δ 164.44, 162.50, 161.76, 158.72, 152.64, 146.86, 140.21, 138.40, 132.78, 131.81, 131.16, 128.25, 123.94, 119.51, 118.08, 117.66, 115.66, 101.16, 96.22, 92.25, 88.69, 57.86, 55.77, 55.10, 53.00, 46.02, 24.45; **HRMS (ESI^+^):** calcd. for C_32_H_32_F_3_N_6_O_2_ (MH^+^) 589.2533, found 589.2531

**Compound 19^1^**

**Compound 20: 5-((8-amino-1,7-naphthyridin-5-yl)ethynyl)-6-methyl-N-(4-((4-methylpiperazin-1-yl)methyl)-3-(trifluoromethyl)phenyl)nicotinamide:** A solution of Bromo compound **7a** (75 mg, 0.16 mmol, 1 equiv), Pd(PPh_3_)_2_ Cl_2_ (10 mol%), CuI (5 mol%) and Triphenylphosphine (10 mg) in Triethylamine (0.65 mL, 4.65 mmol, 30 equiv) was de-oxygenated using steam of Argon gas. A de-oxygenated solution of alkyne **4b** (31mg, 0.19 mmol, 1.2 equiv) in DMF (3 mL) was added slowly over a period of 10 min to the solution and the reaction temperature was increased to 50 ºC and allowed to stir 12 h. The reaction was quenched by addition of NH_4_Cl (5 mL) at room temperature and diluted with ethyl acetate (250 mL). The organic layer was washed with water (5 × 50 mL) and washed with brine (50 mL). Combined organic layers were dried over anhydrous sodium sulphate , filtered and concentrated in vacuo. The pure product **22** was obtained by flash column chromatography. **Yield** = 47%.; **TLC R*_f_*** = 0.2 (10 % MeOH/CH_2_Cl_2_)

**^1^H NMR** (500 MHz, DMSO-*d*_6_) δ 10.68 (s, 1H), 8.94 (d, *J* = 2.3 Hz, 1H), 8.88 (dd, *J* = 4.2, 1.6 Hz, 1H), 8.51 (d, *J* = 2.2 Hz, 1H), 8.47 (dd, *J* = 8.3, 1.6 Hz, 1H), 8.27 (s, 1H), 8.20 (d, *J* = 2.2 Hz, 1H), 8.04 (dd, *J* = 8.5, 2.2 Hz, 1H), 7.72 (d, *J* = 8.5 Hz, 1H), 7.60 (s, 2H), 3.56 (s, 2H), 2.81 (s, 3H), 2.39 (s, 8H), 2.19 (s, 3H); **^13^C NMR** (126 MHz, DMSO) δ 164.07, 162.31, 158.81, 149.79, 148.63, 147.41, 138.39, 137.88, 133.13, 132.79, 132.48, 131.80, 131.35, 127.97, 127.14, 124.00, 118.93, 117.74, 101.58, 92.83, 90.00, 57.84, 55.05, 52.93, 45.94, 24.30; **HRMS (ESI^+^):** calcd. for C_30_H_29_F_3_N_7_O (MH^+^) 560.2386, found 560.2383

**Compound 21: 5-((8-(cyclopropylamino)-1,7-naphthyridin-5-yl)ethynyl)-6-methyl-N-(4-((4-methylpiperazin-1-yl)methyl)-3-(trifluoromethyl)phenyl)nicotinamide:** A solution of Bromo compound 1**5a** (100 mg, 0.38 mmol, 1.2 equiv), Pd(PPh_3_)_4_ (10 mol%), CuI (5 mol%) and Triphenylphosphine (10 mg) in Triethylamine (1.3 mL, 9.3 mmol, 30 equiv) was de-oxygenated using steam of Argon gas. A de-oxygenated solution of alkyne **2b** (130 mg, 0.31 mmol, 1 equiv) in DMF (3 mL) was added slowly over a period of 10 min to the solution and the reaction temperature was increased to 50 ºC and allowed to stir 12 h. The reaction was quenched by addition of NH_4_Cl (5 mL) at room temperature and diluted with ethyl acetate (300 mL). The organic layer was washed with water (5 × 50 mL) and washed with brine (1 × 50 mL). Combined organic layers were dried over anhydrous sodium sulphate, filtered and concentrated in vacuo. The pure product **23** was obtained by flash column chromatography. **Yield** = 66%.; **TLC R*_f_*** = 0.5 (10 % MeOH/CH_2_Cl_2_)

**^1^H NMR** (500 MHz, DMSO-*d*_6_) δ 10.66 (s, 1H), 8.94 (d, *J* = 2.2 Hz, 1H), 8.85 (dd, *J* = 4.3, 1.6 Hz, 1H), 8.50 (d, *J* = 2.4 Hz, 1H), 8.47 (dd, *J* = 8.4, 1.7 Hz, 1H), 8.36 (s, 1H), 8.19 (s, 2H), 8.04 (dd, *J* = 8.5, 2.1 Hz, 1H), 7.85 (dd, *J* = 8.3, 4.2 Hz, 1H), 7.71 (d, *J* = 8.5 Hz, 1H), 3.55 (s, 2H), 3.51 (s, 1H), 2.80 (s, 3H), 2.37 (s, 8H), 2.15 (s, 3H), 0.81 – 0.75 (m, 2H), 0.75 – 0.69 (m, 2H); **^13^C NMR** (126 MHz, DMSO) δ 164.01, 162.33, 157.45, 149.61, 148.37, 147.46, 138.36, 137.82, 133.22, 132.92, 132.83, 131.75, 130.74, 127.91, 127.05, 123.97, 118.85, 117.69, 101.63, 92.79, 90.19, 57.89, 55.16, 53.11, 46.12, 42.51, 24.76, 24.31, 6.89; **HRMS (ESI^+^):** calcd. for C_33_H_33_F_3_N_7_O (MH^+^) 600.2693, found 600.2695

**Compound 22: 3-(isoquinolin-4-ylethynyl)-4-methyl-N-(4-((4-methylpiperazin-1-yl)methyl)-3-(trifluoromethyl)phenyl)benzamide:** A solution of Bromo compound **16a** (100 mg, 0.48 mmol, 1.2 equiv), Pd(PPh_3_)_4_ (10 mol%), CuI (5 mol%) and Triphenylphosphine (10 mg) in Triethylamine (1.3 mL, 9.6 mmol, 20 equiv) was de-oxygenated using steam of Argon gas. A de-oxygenated solution of alkyne **1b** (133 mg, 0.32 mmol, 1 equiv) in DMF (4 mL) was added slowly over a period of 10 min to the solution and the reaction temperature was increased to 50 ºC and allowed to stir 8 h. The reaction was quenched by addition of NH_4_Cl (5 mL) at room temperature and diluted with ethyl acetate (300 mL). The organic layer was washed with water (5 × 50 mL) and washed with brine (1 × 50 mL). Combined organic layers were dried over anhydrous sodium sulphate, filtered and concentrated in vacuo. The pure product **24** was obtained by flash column chromatography. **Yield** = 70%.; **TLC R*_f_*** = 0.5 (10 % MeOH/CH_2_Cl_2_)

**^1^H NMR** (500 MHz, DMSO-*d*_6_) δ 10.56 (s, 1H), 9.37 (s, 1H), 8.82 (s, 1H), 8.33 (d, *J* = 8.8 Hz, 1H), 8.30 (d, *J* = 1.9 Hz, 1H), 8.24 (d, *J* = 8.2 Hz, 1H), 8.21 (d, *J* = 2.2 Hz, 1H), 8.06 (dd, *J* = 8.5, 2.2 Hz, 1H), 8.01 – 7.93 (m, 2H), 7.81 (ddd, *J* = 8.0, 6.9, 1.1 Hz, 1H), 7.70 (d, *J* = 8.5 Hz, 1H), 7.56 (d, *J* = 8.1 Hz, 1H), 3.55 (s, 2H), 2.66 (s, 3H), 2.37 (s, 8H), 2.15 (s, 3H); **^13^C NMR** (126 MHz, DMSO) δ 165.15, 153.18, 146.75, 144.28, 138.62, 134.88, 132.81, 132.59, 131.71, 131.43, 130.54, 129.08, 128.99, 128.93, 127.90, 125.89, 124.69, 124.00, 122.39, 117.75, 115.14, 95.03, 89.39, 57.91, 55.17, 53.12, 46.14, 21.14; **HRMS (ESI^+^):** calcd. for C_32_H_30_F_3_N_4_O (MH^+^) 543.2366, found 543.2369

**Compound 23: 3-((1,7-naphthyridin-5-yl)ethynyl)-4-methyl-N-(4-((4-methylpiperazin-1-yl)methyl)-3-(trifluoromethyl)phenyl)benzamide:** A solution of Bromo compound **17a** (100 mg, 0.48 mmol, 1.5 equiv), Pd(PPh_3_)_4_ (10 mol%), CuI (5 mol%) and Triphenylphosphine (10 mg) in Triethylamine (1.1 mL, 9.6 mmol, 20 equiv) was de-oxygenated using steam of Argon gas. A de-oxygenated solution of alkyne **1b** (133 mg, 0.32 mmol, 1 equiv) in DMF (4 mL) was added slowly over a period of 10 min to the solution and the reaction temperature was increased to 50 ºC and allowed to stir 5 h. The reaction was quenched by addition of NH_4_Cl (5 mL) at room temperature and diluted with ethyl acetate (300 mL). The organic layer was washed with water (5 × 50 mL) and washed with brine (1 × 50 mL). Combined organic layers were dried over anhydrous sodium sulphate, filtered and concentrated in vacuo. The pure product **25** was obtained by flash column chromatography. **Yield** = 78%.; **TLC R*_f_*** = 0.3 (10 % MeOH/CH_2_Cl_2_).

**^1^H NMR** (500 MHz, DMSO-*d*_6_) δ 10.56 (s, 1H), 9.43 (s, 1H), 9.17 (d, *J* = 3.8 Hz, 1H), 8.93 (s, 1H), 8.72 (d, *J* = 9.1 Hz, 1H), 8.32 (s, 1H), 8.20 (s, 1H), 8.06 (d, *J* = 8.5 Hz, 1H), 8.00 – 7.92 (m, 2H), 7.70 (d, *J* = 8.5 Hz, 1H), 7.56 (d, *J* = 8.0 Hz, 1H), 3.55 (s, 2H), 2.65 (s, 3H), 2.38 (s, 8H), 2.16 (s, 3H); **^13^C NMR** (126 MHz, DMSO) δ 165.12, 154.00, 153.78, 147.06, 144.43, 142.40, 138.61, 133.39, 132.83, 132.55, 131.71, 131.63, 131.03, 130.55, 129.26, 127.33, 123.98, 122.08, 117.72, 115.43, 95.59, 88.15, 57.87, 55.11, 53.03, 46.04, 21.12; **HRMS (ESI^+^):** calcd. for C_31_H_29_F_3_N_5_O (MH^+^) 544.2319, found 544.2313

**Compound 24: 5-((1,7-naphthyridin-5-yl)ethynyl)-6-methyl-N-(4-((4-methylpiperazin-1-yl)methyl)-3-(trifluoromethyl)phenyl)nicotinamide:** A solution of Bromo compound 17**a** (65 mg, 0.31 mmol, 1 equiv), Pd(PPh_3_)_4_ (10 mol%), CuI (5 mol%) and Triphenylphosphine (10 mg) in Triethylamine (1.3 mL, 9.3 mmol, 20 equiv) was de-oxygenated using steam of Argon gas. A de-oxygenated solution of alkyne **2b** (130 mg, 0.31 mmol, 1 equiv) in DMF (3 mL) was added slowly over a period of 10 min to the solution and the reaction temperature was increased to 50 ºC and allowed to stir 12 h. The reaction was quenched by addition of NH_4_Cl (5 mL) at room temperature and diluted with ethyl acetate (300 mL). The organic layer was washed with water (5 × 50 mL) and washed with brine (1 × 50 mL). Combined organic layers were dried over anhydrous sodium sulphate, filtered and concentrated in vacuo. The pure product **26** was obtained by flash column chromatography. **Yield** = 49%.; **TLC R*_f_*** = 0.1 (10 % MeOH/CH_2_Cl_2_).

**^1^H NMR** (500 MHz, DMSO-*d*_6_) δ 10.74 (s, 1H), 9.46 (s, 1H), 9.18 (dd, *J* = 4.2, 1.6 Hz, 1H), 9.03 (d, *J* = 2.3 Hz, 1H), 8.96 (s, 1H), 8.76 (d, *J* = 8.3 Hz, 1H), 8.65 (d, *J* = 2.3 Hz, 1H), 8.20 (d, *J* = 2.2 Hz, 1H), 8.05 (dd, *J* = 8.5, 2.2 Hz, 1H), 7.97 (dd, *J* = 8.5, 4.1 Hz, 1H), 7.72 (d, *J* = 8.5 Hz, 1H), 3.57 (s, 2H), 2.86 (s, 3H), 2.40 (s, 8H), 2.20 (s, 3H); **^13^C NMR** (126 MHz, DMSO) δ 163.86, 163.17, 154.38, 153.87, 148.62, 147.27, 142.36, 139.00, 138.34, 133.44, 132.83, 131.81, 131.07, 128.05, 127.41, 124.00, 117.69, 115.01, 93.66, 90.16, 57.82, 55.02, 52.87, 45.87, 24.31; **HRMS (ESI^+^):** calcd. for C_30_H_28_F_3_N_6_O (MH^+^) 545.2271, found 545.2269

**4. References:**

1. Larocque, E.; Naganna, N.; Ma, X.; Opoku-Temeng, C.; Carter-Cooper, B.; Chopra, G.; Lapidus, R. G.; Sintim, H. O., Aminoisoquinoline benzamides, FLT3 and Src-family kinase inhibitors, potently inhibit proliferation of acute myeloid leukemia cell lines. *Future Med Chem* **2017,** *9* (11), 1213-1225.

1. **HPLC Traces of Compounds**

**Conditions**: Agilent Eclipse plus C18 column, 3,5 µm, 4.6×100 mm, 0→15 min, 50% B→100% B(A: 0.1% NH4OH in H_2_O, B: MeOH), 50 ^o^C.


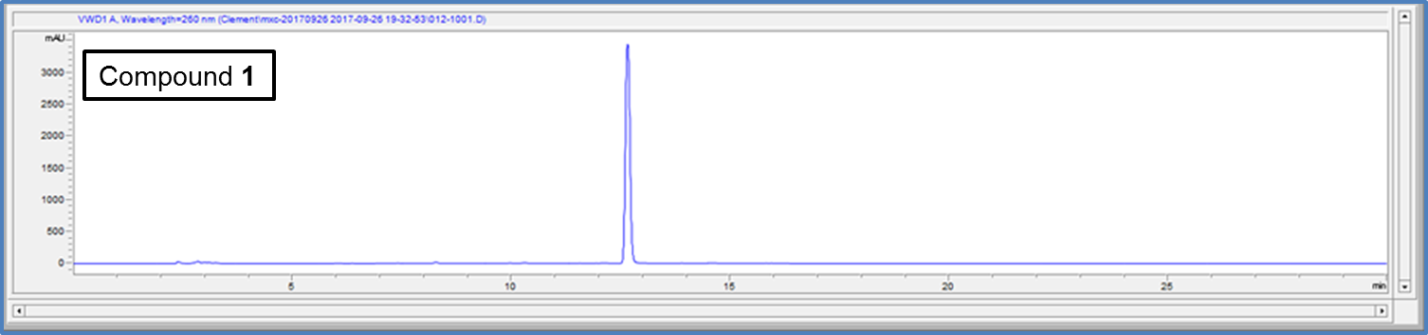


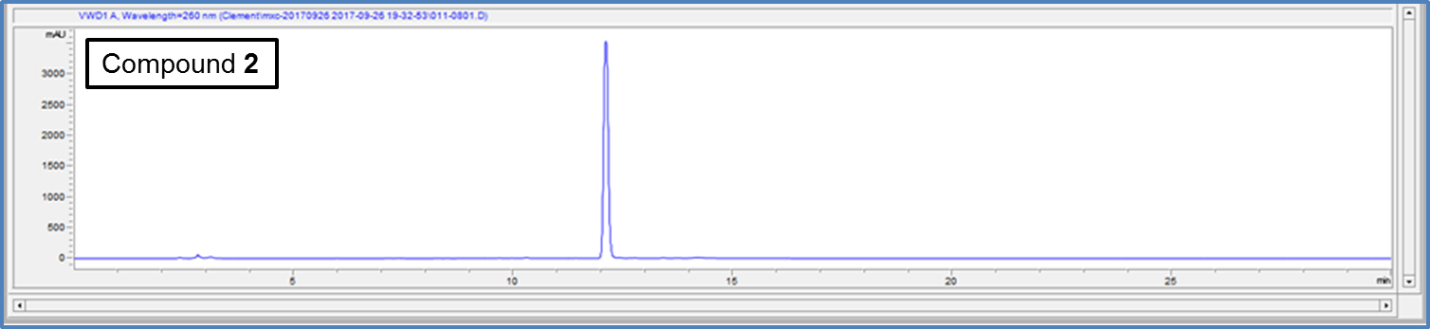


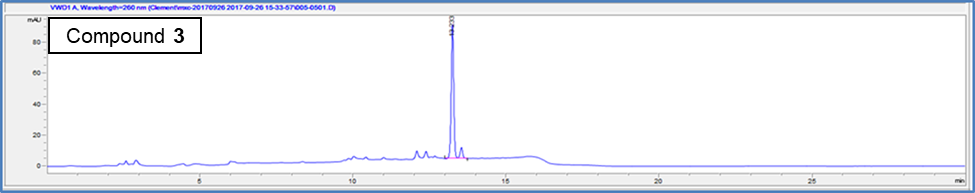


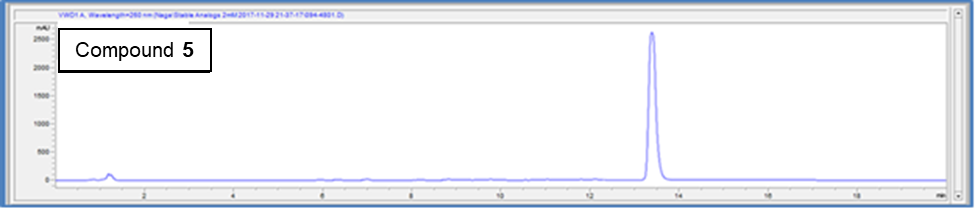


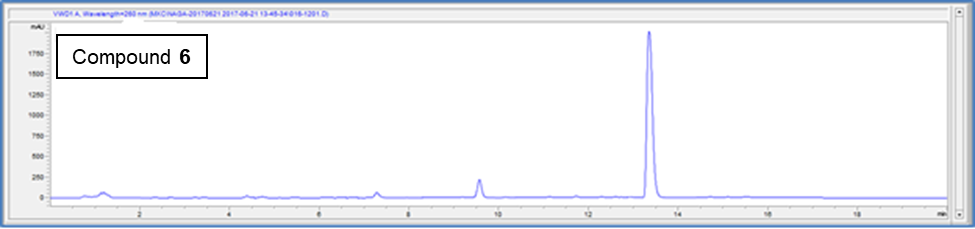


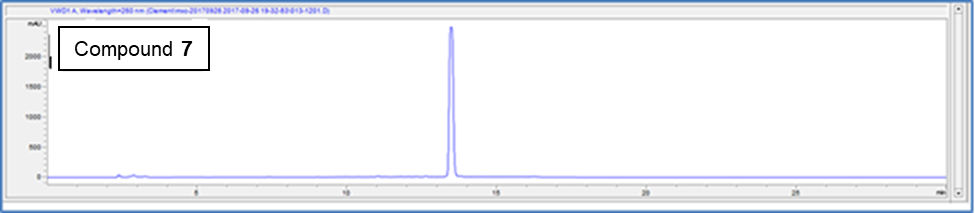


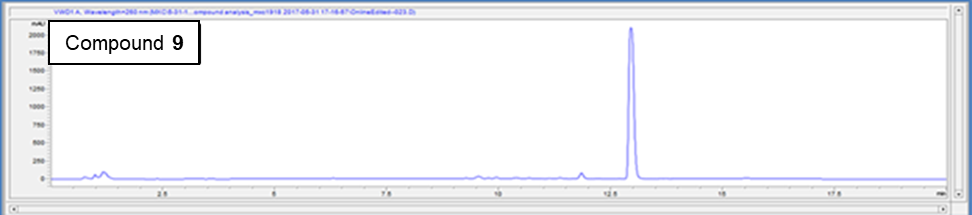


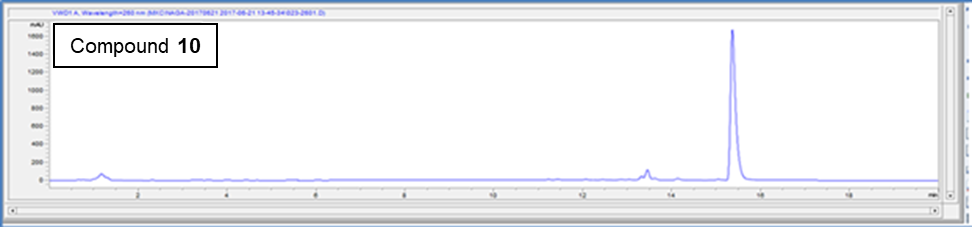


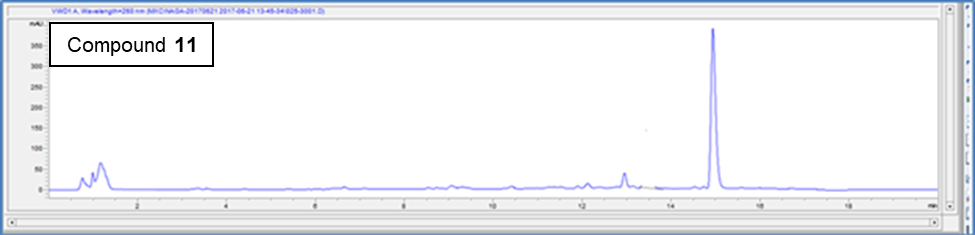


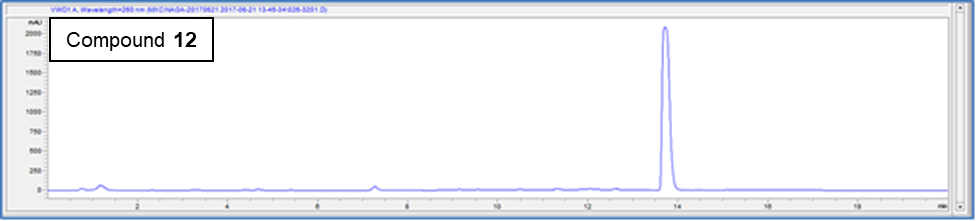


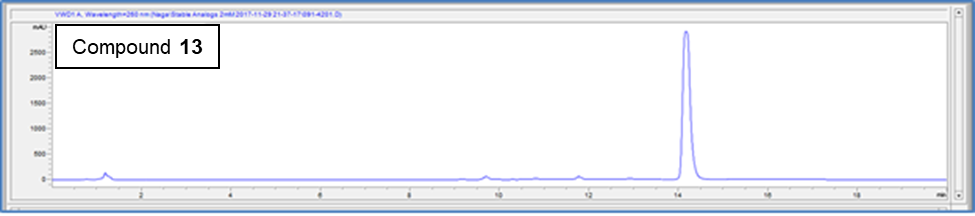


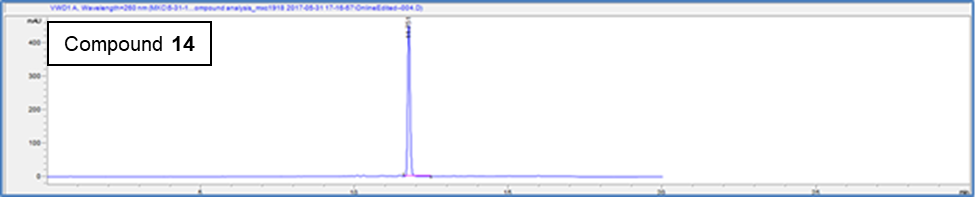


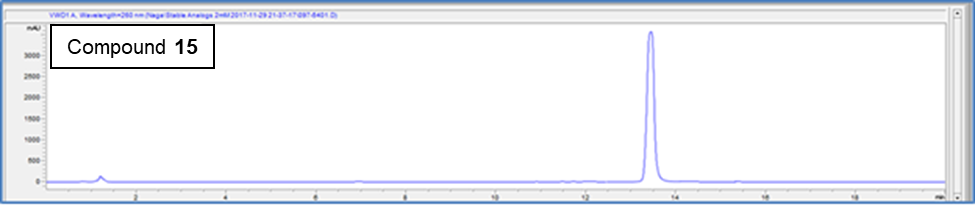


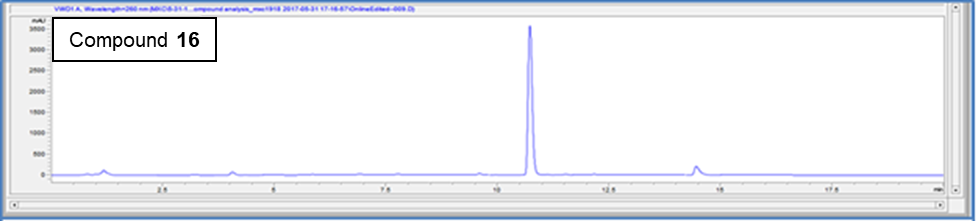


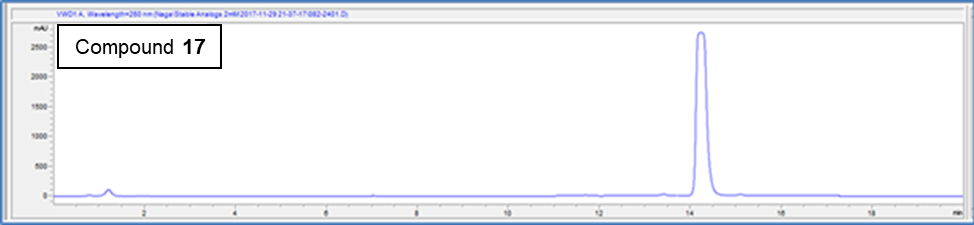


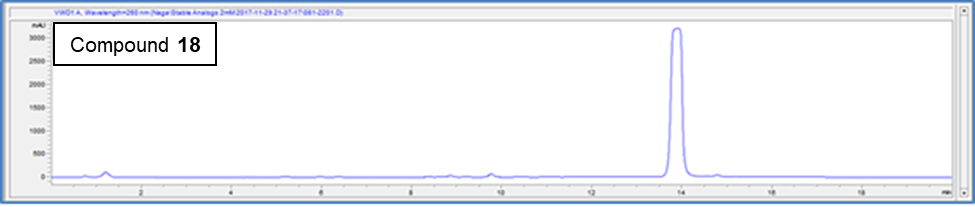


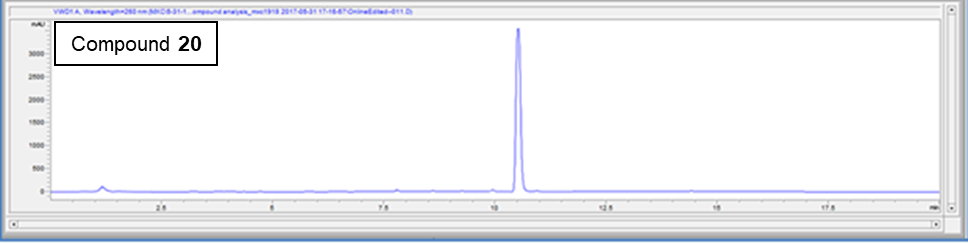


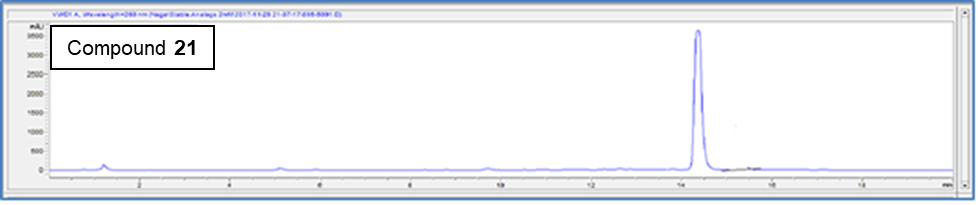


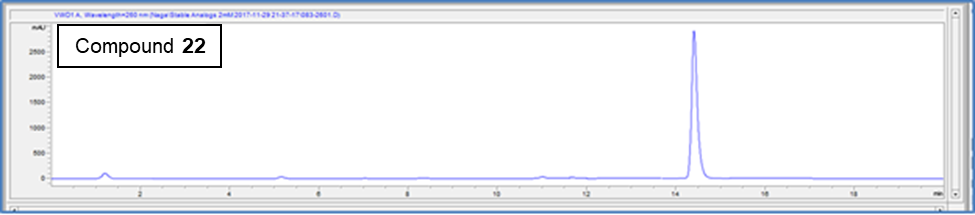


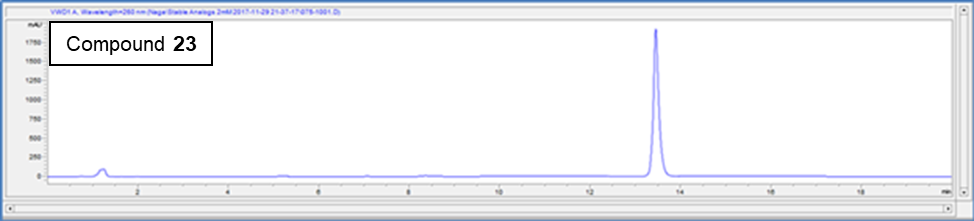


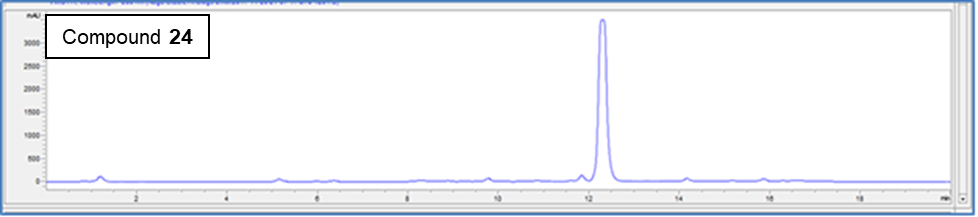


1. **NMR Spectra of Compounds**

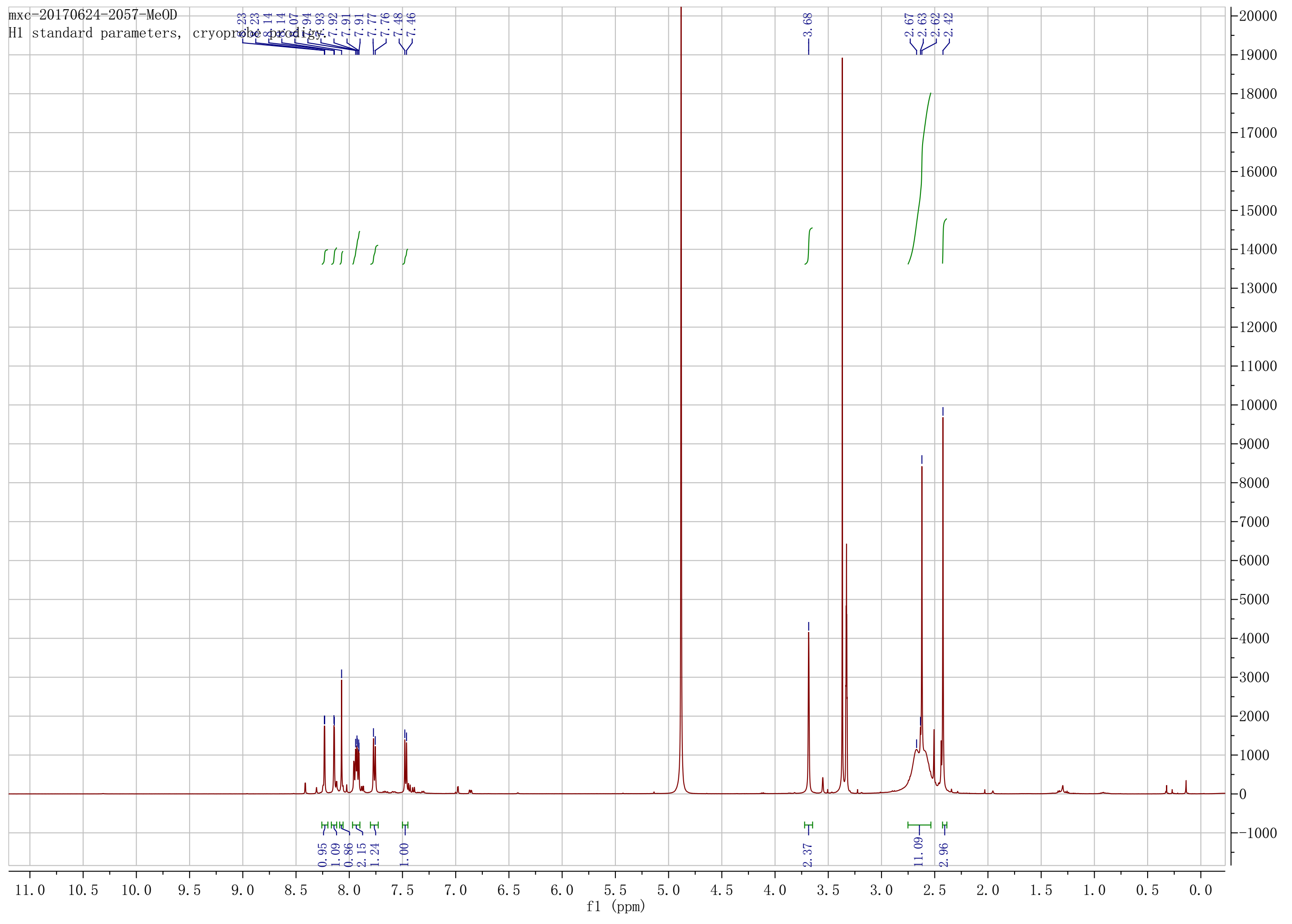

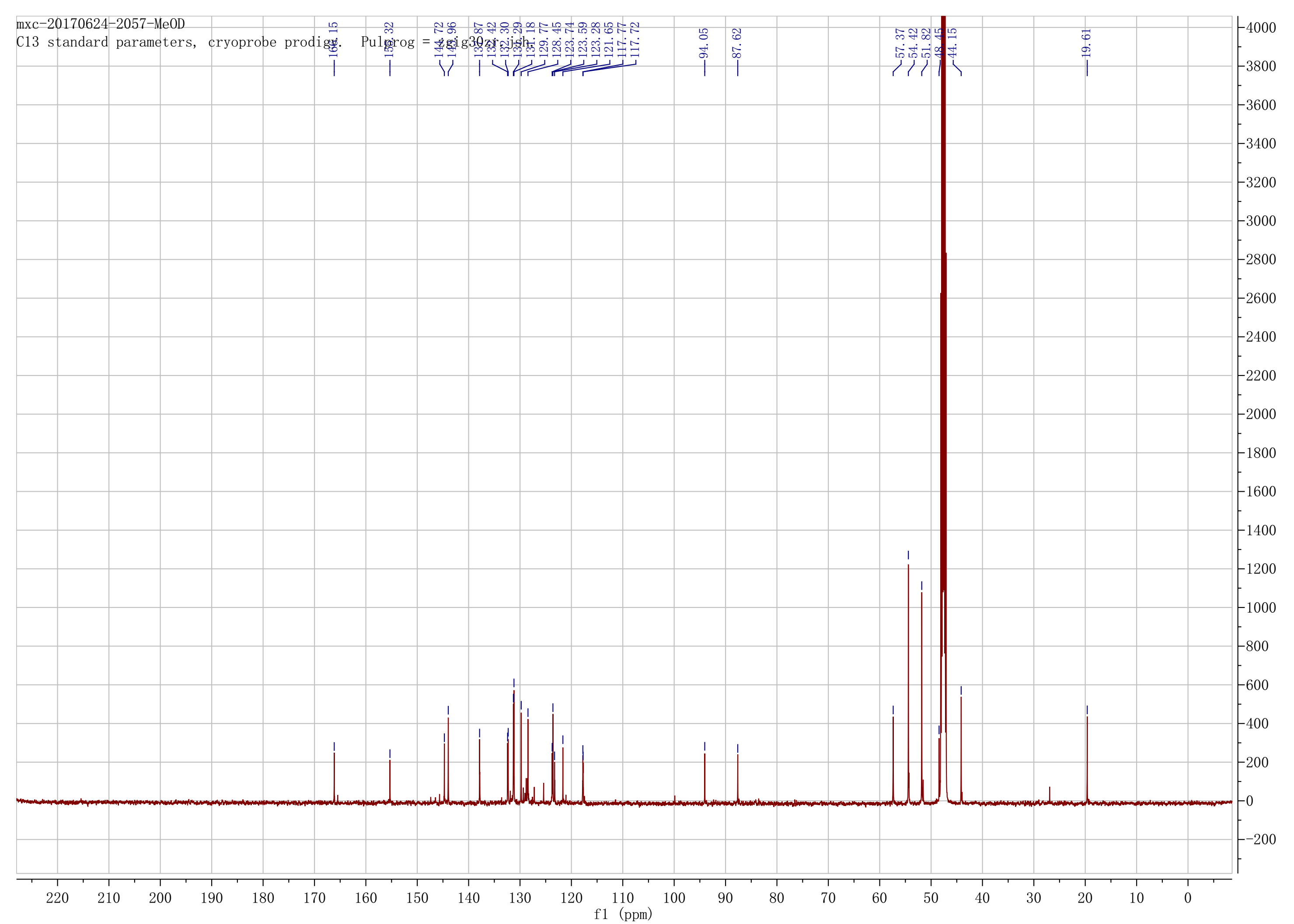

Supplement: Supplementary file 1 — Supplementary material [file mmc1.docx]
